# Quantum Mechanical/Molecular Mechanical Analysis of the Catalytic Mechanism of Phosphoserine Phosphatase

**DOI:** 10.3390/molecules23123342

**Published:** 2018-12-17

**Authors:** Dieter Krachtus, Jeremy C. Smith, Petra Imhof

**Affiliations:** 1 Computational Molecular Biophysics Group, Interdisciplinary Center for Scientific Computing (IWR), Im Neuenheimer Feld 368, 69120 Heidelberg, Germany; dieter.krachtus@genflux.de (D.K.); smithjc@ornl.gov (J.C.S.); 2 Oak Ridge National Laboratory, Center for Molecular Biophysics, University of Tennessee, One Bethel Valley Road, P.O. Box 2008, Oak Ridge, TN 37831-6255, USA; 3 Institute for Theoretical Physics, Freie Universität Berlin, Arnimallee 14, 14195 Berlin, Germany

**Keywords:** phosphoserine phosphatase, QM/MM, reaction pathways, reaction coordinate

## Abstract

Phosphoserine phosphatase (PSP), a member of the haloacid dehalogenase (HAD) superfamily that comprises the vast majority of phosphotransferases, is likely a steady-state regulator of the level of d-serine in the brain. The proposed catalytic cycle of PSP consists of a two-step mechanism: formation of a phospho-enzyme intermediate by phosphate transfer to Asp11 and its subsequent hydrolysis. Our combined quantum mechanical/molecular mechanical (QM/MM) calculations of the reaction pathways favour a dissociative mechanism of nucleophilic substitution via a trigonal-planar metaphosphate-like configuration for both steps, associated with proton transfer to the leaving group or from the nucleophile. This proton transfer is facilitated by active site residue Asp13 that acts as both a general base and a general acid. Free energy calculation on the reaction pathways further support the structural role of the enzymatic environment and the active site architecture. The choice of a proper reaction coordinate along which to bias the free energy calculations can be guided by a projection of the canonical reaction coordinate obtained from a chain-of-state optimisation onto important internal coordinates.

## 1. Introduction

**Figure 1:**
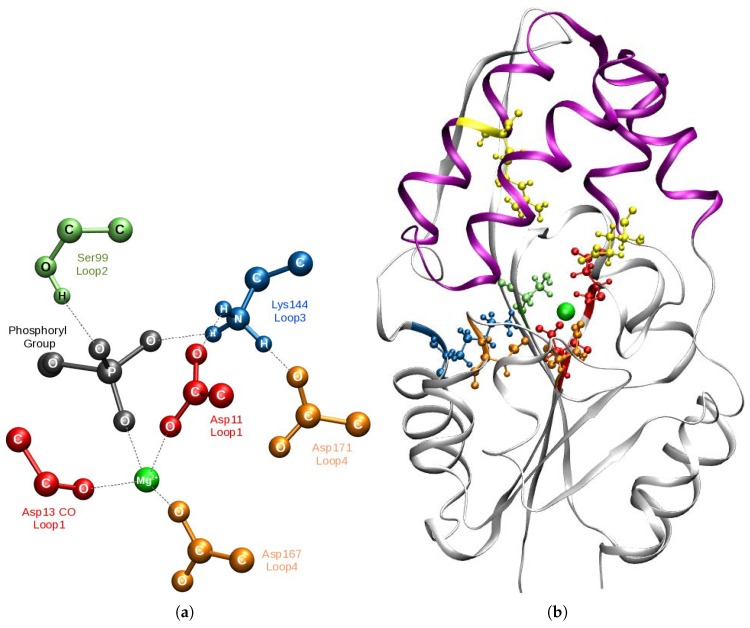
Schematic structural overview of PSP and HAD I superfamily members: (**a**) Catalytic scaffold of PSP comprised of four loops. The backbone carbonyl oxygen of Loop 1 (red) and the carboxyl groups of Loop 4 (orange) bind the Mg${}^{2+}$ cofactor. The carboxyl group of Asp11 of Loop 1 acts as nucleophile. Loop 2 (lime) and Loop 3 (blue) position the nucleophile and substrate phosphoryl group (black). (**b**) Ribbon diagram of PSP. α/β core domain (white); inserted between Loops 1 and 2 of the core domain is the α-helical-bundle cap domain (violet) that completes the catalytic scaffold by supporting residues Glu20 and Arg56 (Loop 5, yellow). Substrate phospho-l-serine omitted.

Enzymatic phosphoryl group transfer is found at the core of many cellular biochemical reactions [1]. Phosphate monoester hydrolysis is vital in regulating processes such as DNA replication [2], gene transcription and translation [3,4] and apoptosis [5]. The consistently low rate of this reaction in aqueous solution [6] is enhanced by up to ∼$10{}^{21}$ by the catalytic action of phosphotransferases. These enzymes lower the kinetic barrier through general acid–base catalysis, nucleophilic catalysis and by strongly stabilizing (K${}_{d}$∼$10{}^{-26}$ M) the reaction intermediates [6] through an ensemble of electrostatic and steric interactions [7,8,9]. The specificity of these interactions is vital for phosphoryl group transfer as exemplified by the independent co-evolution of a strikingly similar catalytic scaffold (see Figure 1) in many families of phosphotransferases belonging to the haloacid dehalogenase-like hydrolase (HAD) superfamily [10], comprising phosphatases, phosphomutases, and P-type ATPases. As an absolute catalytic requirement, the scaffold of HAD family members has a consensus sequence for a metal-binding motif that holds the divalent Mg${}^{2+}$ ion.

There is much debate about the nature of the transition state of HAD family members [11,12,13] and whether the nucleophilic-substitution mechanism has either dissociative or associative characteristics (or somewhere between the two extremes) [9,14,15]. The nature of the transition state of a phosphoryl transfer reaction is determined by the difference in timing of the formation of the phosphorous-nucleophile bond and cleavage of the phosphorous leaving-group bond. The transition state of an associative mechanism is thought to be a bi-pyramidal phosphophorane-like pentavalent species, whereas a dissociative mechanism shows a trigonal-planar metaphosphate-like species [11,12,13].

Phosphoserine phosphatase (PSP) is an important enzyme in the (de-)phosphorylation pathway of serine biosynthesis. The main portion of the endogenous l-serine level is contributed by PSP [16,17] which is converted to d-serine [18,19], a co-agonist of the *N*-methyl-d-aspartate (NMDA) subtype of glutamate neurotransmitter receptors [20,21].

The enzymatic phosphate transfer reaction of PSP is Mg${}^{2+}$ metal ion dependent. It has been shown that for human PSP the presence of a Ca${}^{2+}$ ion in the active site of the enzyme inhibits its catalytic activity even in the presence of Mg${}^{2+}$ [22]. It has been argued that this inhibition is due to the sevenfold coordinated nature of Ca${}^{2+}$, which therefore captures both oxygen atoms of the nucleophilic aspartate residue, thus hampering the attack by the aspartate at the phosphorus atom of the substrate PLS.

PSP is a member of the first of the three HAD subfamilies (I–III) distinguished by the presence (I and II) and location of a second domain that functions as a cap over the active site α/β core domain [23,24] found in all members of the superfamily. The core domain consists of a central parallel β-sheet flanked by α-helices supporting four loops which comprise the catalytic scaffold (see Figure 1) [25]. The cap domain of subfamily I completes the active site by closing the catalytic site to solvent in response to substrate binding and in supporting chemical diversification by contributing tailored residues (Loop 5) that provide stereospecificity and orientation to the specific phosphoryl substrate being cleaved (see Figure 1) [26,27,28].

**Figure 2:**
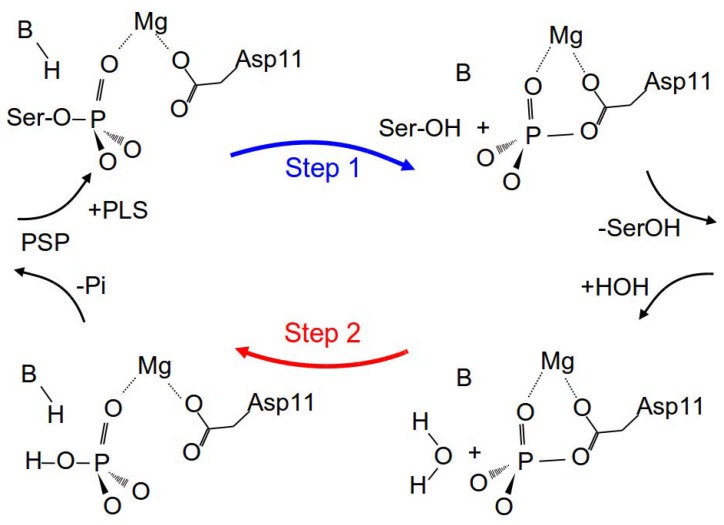
Reaction scheme of the two steps in the proposed mechanism of the phosphate transfer catalysed by phosphoserine phosphatase PSP. Phospho-l-serine (PLS) is bound by the enzyme. Then, the phosphate group is in a first step transferred to Asp11, with the help of a residue B, acting as general acid. Serine leaves the enzyme. In a second step, the phosphoryl-aspartate is hydrolysed, again assisted by residue B, now acting as general base. Figure adapted from Ref. [26]

High-resolution experimental and modelled structures of PSP from *Methanococcus jannaschii* have been constructed to help understand the catalytic cycle of PSP [26,29,30]. The catalytic cycle consists of two phosphate transfer steps. Step 1 is the actual dephosphorylation of phosph-l-serlin (PLS) and formation of a phosphoryl-aspartate intermediate (see Figure 2). In Step 2, the hydrolysis of the phospho-enzyme intermediate, the PLS substrate is replaced by a water molecule. The crystal structures reported in [26] represent the reactant state, a D11N mutant structure bound to PLS without the metal cofactor Mg${}^{2+}$, a PSP + BeF${}_{3}^{-}$ complex mimicking the phospho-enzyme intermediate, a transition-state structural analogue with the PSP complexed to AlF${}_{3}$, and the product state of the enzyme with inorganic phosphate in the active site. From these structures, the reaction mechanisms of the two steps were suggested to proceed via an associative transition state on the grounds of the nucleophile-phosphorus (Aluminium) atom distances of 3.59 Å (Asp11 $O_{\delta 1}$–P) in Step 1 and 3.56 Å in Step 2 (H${}_{2}$O–Al), which were much smaller than the ∼4.9 Å of a fully dissociative mechanism [31]. However, it was emphasized that the geometries and charge distributions of the BeF${}_{3}^{-}$ and AlF${}_{3}$ complexes are probably different from the real transition states and that further studies are necessary to investigate the transition states directly to elucidate the reaction mechanism [26].

Hybrid quantum mechanical/molecular mechanical (QM/MM) studies, with different levels of theory for the QM part, have in the past proven successful in the analysis of enzymatic phosphate transfer reactions [13,32,33,34,35,36,37,38,39,40,41,42,43,44,45,46,47,48,49,50,51,52]. For Step 1 of the phosphate transfer reaction by PSP, Re et al. [53] performed QM/MM calculations employing density functional theory (B3LYP/6-31G+(d)). When computing one-dimensional (along the linear combination of the forming and breaking P–O bond) and two-dimensional potential energy scans, the latter also including a proton transfer coordinate, they found the latter approach more suitable to describe the reaction mechanism due to the importance of a proton transfer from residue Asp13 to the oxygen atom of the leaving group serine. The computed mechanism is clearly dissociative with a metaphosphate-like transition state whose P–O distances are larger than the Al–O distances in the transition state analogue complex [26], although still shorter than those in a fully dissociative mechanism [31]. The barrier computed along their mechanism (4.0 kcal/mol) is considerably lower than uncatalysed phosphate transfer reactions which range between ∼35–40 kcal/mol [11,34], suggesting a tremendous catalytic effect by the enzyme. The experimental rate k${}_{\mathrm{cat},PSP}$ = 20 min${}^{-1}$ measured at ∼$70{\;}^{\circ }$C [26] translates to a barrier of ∼21 kcal/mol according to transition state theory. With a very low barrier for the first phosphate transfer step, Step 2, i.e., the hydrolysis of the phosphoryl-aspartate, is likely the rate determining step of the two (disregarding substrate binding and product release).

To explore this possibility and to further examine the reaction mechanism and the associative or dissociative character nature of the transition state species, we report here on QM/MM simulations of the two phosphoryl-transfer steps, computed on the potential energy landscape and augmented by free energy calculations. Special attention is paid to the choice of reaction coordinates, confirming the previously observed [53] importance of explicitly accounting for proton transfer events. These have a catalytic impact by assisting in the departure of the leaving group in Step 1 and the activation of the nucleophilic water molecule attacking at the scissile phosphorus atom in Step 2.

## 2. Materials and Methods

The reactants of Step 1 and Step 2 were modelled using the crystal structures of the PLS-bound D11N mutant, the transition state analogue PSP-AlF${}_{3}$ complex and the phosphoryl-intermediate mimic, the PSP-BeF${}_{3}$ complex with PDB codes 1L7P, 1L7N and 1J97, respectively [26,29,30]. For the reactant state of Step 1 (1R), the PLS substrate was modelled into 1L7N using coordinates from 1L7P. For the reactant of Step 2 (2R), the BeF${}_{3}^{-}$ analogue was replaced by a phosphate group. The reactant of Step 1 (1R) was set up in two different protonation states: (1) dianionic phosphoserine and neutral Asp11; and (2) anionic phosphoserine and negatively charged Asp11.

### 2.1. Active Site Model Calculations

To evaluate the QM method to be used in the QM/MM calculations, purely quantum mechanical calculations of minimal active site models were performed for the two steps of the phosphate transfer reaction in PSP (cf. Figures S2 and S3).

The minimal model consists of methyl-phosphate to mimic the substrate PLS, the metal cofactor Mg${}^{2+}$, an acetate ion representing Asp11, formaldehyde as a model for the backbone of Asp13, and three water molecules to saturate the sixfold coordination of the Mg${}^{2+}$ ion. Calculations with the minimal model were performed at the following two levels of theory and compared:

(1) Density functional theory (DFT), applying Becke’s three-parameter hybrid functional, B3LYP, with a 6-31++G(d,p) basis set. The DFT calculations were performed with the Turbomole program package [54]. Geometries of stationary points were optimized to a gradient of 10${}^{-4}$$E_{H}/bohr$. The nature of the stationary point (minimum or transition state) was verified by normal mode analysis at the optimized geometries.

(2) The density functional tight binding method, DFTB3/3OB, using specific parameters for phosphorus and magnesium, was employed to represent the QM region [55,56,57], which has proven a good approximation to computationally more expensive DFT calculations for phosphate hydrolysis reactions in enzymes [33,58] and cluster models [57]. All DFTB calculations were performed using CHARMM [59] interfaced to SCC-DFTB [55,56].

Minima for the reactant, product, and intermediate states were obtained with 10^−7^ a.u. as the SCF convergence criterion and 10^−4^ a.u./Å as the criterion for the geometry optimization. Transition states between minima were found by using the Conjugate Peak Refinement [60] algorithm as implemented in CHARMM [59] that identifies a minimum energy path between two given (minima) end states.

For all optimized geometries, energies were further evaluated with single-point calculations at the B3LYP/6-31++G(d,p) level of theory.

### 2.2. MD Simulation

Short molecular dynamics simulations were performed for the reactant state of Step 1, 1R, in two different protonation states (anionic and dianionic PLS) and for the reactant state of Step 2, 2R, using the program NAMD [61], applying the CHARMM27 force field [59]. Each protein model was solvated in explicit water extending 15 Å in each direction in a cubic box (x = 90 Å, y = 90 Å, z = 90 Å), applying periodic boundary conditions. The system was neutralized by adding 16 Cl${}^{-}$ and 14 Na${}^{+}$ counter ions, added by random substitution of water oxygen atoms.

Long-range electrostatic interactions were computed using the Particle Mesh Ewald method [62] on a 90 Å × 90 Å × 90 Å grid, with a non-bonded cutoff at 10 Å and a smoothing function at 8 Å and 10 Å cutoff applied for short-range electrostatic and van der Waals interactions, respectively. The system was energy minimized and subsequently heated by increasing the temperature in steps of 1 K from 0 to 300 K during 30 ps with random reassignment of velocities every 0.1 ps, and then equilibrated with the numbers of particles, pressure (1.01325 bar) and temperature kept constant during 3 × 25 ps equilibration time, applying Langevin dynamics to maintain constant temperature and Nosé–Hoover Langevin piston with a decay period of 500 fs for constant pressure [63,64]. In the three equilibration steps, harmonic constraints were applied and were gradually lifted (0.5, 0.25 and 0.05 kcal·mol${}^{-1}\cdot$Å${}^{-2}$). An integration time step of 2 fs was used and coordinates were saved with a sampling interval of 2 ps. The SHAKE algorithm [65] was applied to constrain covalent bonds with hydrogen atoms. For each of the two states, 1 ns production run was performed in the NPT ensemble at 1.01325 bar pressure and 300 K.

### 2.3. QM/MM Pathway Calculations

For the QM/MM calculations, all water molecules further than 5 Å from the protein were deleted. The outer part of the system, i.e., all atoms further than 5 Å from the quantum-mechanically treated atoms, was kept fixed. This serves as a compromise between including part of the solvation shell in the enzymatic system without the need for its explicit sampling along the reaction pathways. This allows the potential energy scan, minimum energy pathway calculations, and umbrella sampling molecular dynamics simulations to be performed with the same system setup.

**Figure 3:**
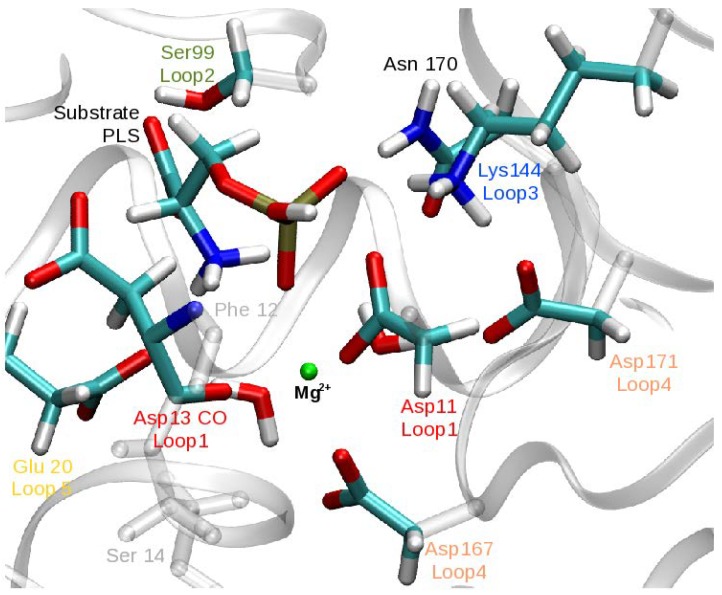
Atoms comprising the larger QM region as described in Section 2.3. The small QM region differs from the large QM region in the exclusion of the atoms of residues Asn170 and Asp171.

The following part of the system was treated quantum mechanically: the Mg${}^{2+}$ ion, four metal-bound water molecules, phosphoserine (PLS) or the nucleophilic water molecule in Step 1 and Step 2, respectively, the side chains of Asp11, Glu20, Ser99, Lys144, Asp167, Asp13 and the link atoms placed at the QM/MM boundary. The size of the QM region was validated by pathway calculations with a larger region that also comprises the side chains of Asn170 and Asp171 (Figure 3). The results of this comparison show that pathways were structurally and mechanistically very similar and the energy profiles were reproducible within ∼2 kcal/mol, independent of the size of the QM region (see Table S3 in the Supplementary Materials). All subsequent QM/MM calculations (in particular free energy calculations, see below) were carried out only with the smaller QM region.

All QM/MM calculations were performed using CHARMM [59] interfaced to SCC-DFTB [55,56] as quantum method. Comparison of a phosphate hydrolysis in a minimal active site model computed with DFTB and DFT (B3LYP/6-31++G(d,p) ) show good agreement (see Tables S1 and S2 in the Supplementary Materials), rendering the semi-empirical method suitable for the present task.

The molecular mechanical part of the system was represented by the CHARMM27 [66] force field and the TIP3P [67,68] water model. The QM/MM boundary was treated with the link atom approach [69].

#### 2.3.1. Minimum Energy Pathways and Potential Energy Scans

Reaction pathways and the associated transition states were initially determined using the Conjugate Peak Refinement (CPR) [60] algorithm as implemented in CHARMM [59]. Pathways obtained by CPR were subsequently refined by adding interpolation points so as to have an equidistanc spacing of 0.01 Å. All non-stationary points along the pathway were further optimised by synchronuous chain minimisation to a tolerance of the projected gradient of 0.1 kcal·mol${}^{-2}$/Å.

For the actual phosphate transfer steps, we performed one- and two-dimensional scans of the potential energy landscape. The one-dimensional scans were carried out along the linear combination of the breaking and forming phosphorous-oxygen bonds, that is RC = [d(SerOG–P) − d(P–OD-Asp11)] for the first and RC = [−d(watO–P) + d(P–OD-Asp11)] for the second step, respectively. The two-dimensional scans were carried with two different sets of combined reaction coordinates. One set comprises both the breaking and forming phosphorous—oxygen distances as RC1 = d(SerOG–P) and RC2 = d(P–OD-Asp11) for Step 1 and RC1 = d(P–OD-Asp11) and RC2=d(watO–P) for Step 2 of the enzymatic reaction. The other set consists of the same combination used for the one-dimensional scan (RC1 = [d(SerOG–P) − d(P–OD-Asp11)] and RC = [−d(watO–P) + d(P–OD-Asp11)] for Step 1 and Step 2, respectively) with an additional coordinate composed of proton-oxygen distances to consider the proton transfer associated with the P–O bond breaking/formation separately. These coordinates are RC2 = [−d(SerOG–H) + d(H–OD-Asp13)] for the first and RC2 = [d(watO–H) − d(H–OT-Asp11)] for the second step, respectively. OT indicates a non-bridging oxygen atom of the phosphate group on Asp11.

The distances defining the reaction coordinates were harmonically restrained with a force constant of 1000 kcal· mol${}^{-1}$·Å${}^{-2}$ and scanned with a step-size of 0.1 Å. All degrees of freedom of the flexible atoms orthogonal to these reaction coordinates were then optimised to a gradient of 0.001 kcal· moll${}^{-1}$· Å${}^{-1}$.

#### 2.3.2. Umbrella Sampling Simulations

To obtain free energy profiles for both steps of the phosphate transfer reaction pathway, we performed umbrella sampling molecular dynamics simulations. To this end, for each of the grid points from the constrained optimisation in the one- or two-dimensional potential energy scans, we performed a QM/MM MD simulation biasing the system to the respective values of the reaction coordinate(s) with a harmonic potential with a force constant of 1000 kcal· mol${}^{-1}\cdot$Å${}^{-2}$ for equilibration and a force constant of 200 kcal· mol${}^{-1}\cdot$Å${}^{-2}$ in the production runs (see below).

In these umbrella sampling MD simulations the system was initially equilibrated for 10 ps by velocity rescaling. Then, 30 ps MD simulations were performed in the NVT ensemble in which temperature was controlled at 300 K by a Nosé–Hoover Langevin thermostat [70]. Another 60 ps simulation time was then used as the production run while monitoring the actual values of the reaction coordinates for every time step. The integration step was 1 fs.

#### 2.3.3. Analysis

The free energy landscape (potential of mean force) was obtained from the umbrella sampling simulation data by employing the Weighted Histogram Analysis Method (WHAM) [71,72] to connect the probability distributions at the separate grid points.

Minimum (free) energy pathways on the two-dimensional potential and free-energy landscapes were computed by converting the two-dimensional grid of potential or free energy values into a simple weighted graph, in which the grid points represent the nodes and each node has an edge to its next neighbours with the higher of the two energies used as the edge weight. The shortest path between the two nodes representing the local minima of reactant and product state was then computed using Dijkstra’s algorithm [73] on the simple weighted graph.

Error estimates were obtained from block-averaging. To this end, the umbrella sampling MD data were partitioned into three blocks, each of 20 ps simulation time. A potential of mean force was then computed for each of these blocks. The standard deviation from the mean is given as the error estimate.

## 3. Results

### 3.1. Enzymatic Reactant State Structure 1R

**Figure 4:**
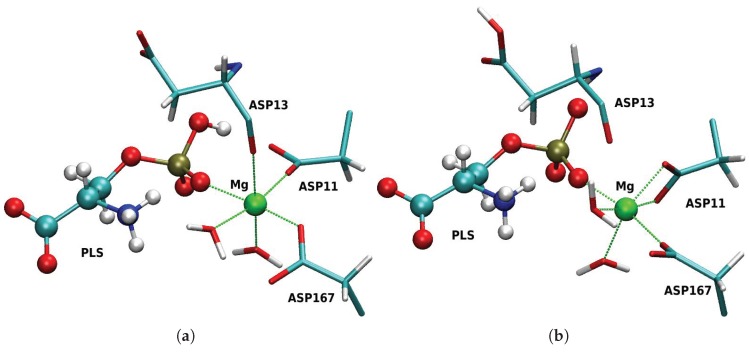
Snapshots from the MD simulation of the reactant state of the first phosphoryl transfer in PSP with different protonation at the scissile phosphate group: (**a**) Anionic phosphoserine. The Mg${}^{2+}$ ion is coordinated by PLS, Asp167, one carboxyl oxygen atom of Asp11 and the backbone oxygen atom of Asp13. (**b**) Dianionic phosphoserine. The Mg${}^{2+}$ ion coordination by the backbone oxygen atom of Asp13 is replaced by the second carboxyl oxygen atom of Asp11.

The active site conformation of PSP depends on the protonation state of the substrate, phospho-l-serine (PLS), as does the composition of the coordination sphere of the central magnesium ion. Figure 4 shows snapshots from the MD simulation of the reactant model in the two PLS protonation states. In the case of anionic PLS (Figure 4a), the active site conformation resembles the crystal structure [26]: the magnesium ion is coordinated by the phosphate group, one carboxyl oxygen of Asp11 and Asp167, the backbone carbonyl oxygen of Asp13 and two water molecules. Asp11 is oriented in-line for a nucleophilic attack at the phosphorous atom. In contrast, in the course of the simulation with dianionic PLS (Figure 4b), Asp11 and the double-negatively charged phosphate group repel each other and Asp11 changes its conformation such that the carboxyl oxygen atom leaves its pre-attack position and instead ligates the magnesium ion. This Asp11 ligand substitutes the carbonyl oxygen atom of Asp13 which is >3.5 Å from the magnesium ion (see Figure 4). The conformational change of Asp11 into a position unfavourable for attack means that the state with dianionic PLS is unlikely to be catalytically relevant.

### 3.2. QM/MM Minimum Energy Pathways

#### 3.2.1. Reaction Path of Step 1: Phosphate Transfer from Serine to Aspartate 11

**Figure 5:**
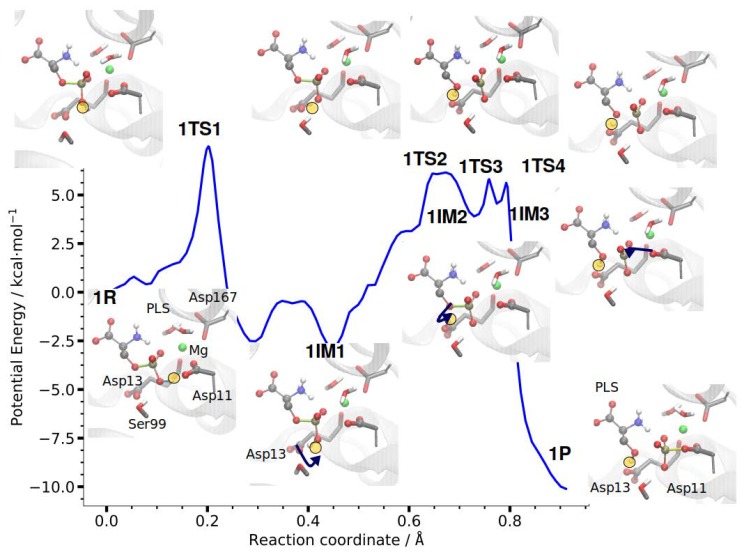
DFTB/MM energy profile of the first phosphate transfer step in PSP. The insets show active site residues of the reactant state, 1R, intermediate states, 1IM1, 1IM2, and 1IM3, and product state, 1P, as well as the transition states in between. The transferred protons are highlighted in yellow, P-O bonds formed or broken in the course of the reaction are also coloured yellow. Arrows indicate the transitions: First, the proton of the phosphate group rotates towards the oxygen atom of Asp13 (1R to 1IM1). Then, this proton is transferred to Asp13 (1IM1 to 1IM2) A further proton transfer from Asp13 to the leaving group oxygen atom (1IM2 to 1IM3) is accompanied by the dissociation of the P–O bond. The resulting high-energy intermediate is attacked by Asp11 resulting in the formation of the phosphoryl-aspartate (1P).

The first dephosphorylation step in PSP, phosphate transfer from PLS to Asp11 (Figure 5), proceeds along the (QM/MM) computed minimum energy pathway with rather small atomic displacements. First, the OH group of the scissile phosphate group rotates towards Asp13 during which the hydrogen bond from Ser99-OH to the phosphate OH group transiently breaks. The corresponding transition state, 1TS1, is the highest energy point (7.5 kcal/mol) along the profile. Next, the proton is transferred (1TS2 with 6.1 kcal/mol energy relative to the reactant) from the phosphate group to the side chain of Asp13 (1IM2). The proton orients and then transfers to the O${}_{\gamma }$ atom of the leaving-group serine, forming an intermediate (1IM3 with 4.6 kcal/mol) not much lower in energy than the transition states leading to 1TS3 (5.8 kcal/mol) and from it 1TS4 (5.6 kcal/mol). After dissociation of the P–O bond the nucleophilic attack of the Asp11 carboxyl oxygen $O_{\delta 1}$ leads to formation of the phosphoryl-aspartate 1P. The transition state of this final phosphate transfer (1TS3) is clearly dissociative with both P–O distances larger than 2.2 Å.

Catalytically, the reactions of Step 1 can be summarized as:

First, Asp13, located at Loop 1 of the catalytic scaffold, acts as general base by accepting a proton from the approaching anionic phosphate group of the substrate PLS. Ser99, a conserved residue of Loop 2 [25] forms hydrogen bonds with the phosphate OH, helping to place and orientate the substrate PLS. However, this hydrogen bond has to be broken during the proton transfer to Asp13, likely leading to an increase in the associated energy barrier. The reformed hydrogen bond, then likely contributes in stabilising the resulting dianionic PLS with additional help by the Mg${}^{2+}$-ion and positively-charged core domain residues (Arg56, Lys144).

Second, Asp13 acts as general acid by transferring the received proton and thereby facilitating leaving group departure. Consequently, P-O${}_{\gamma }$ bond cleavage is observed prior to nucleophilic attack.

Furthermore, Glu20 (and Arg56), both supported by Loop 5 of the cap domain of PSP (Figure 1), provide substrate orientation and stereospecifity by binding the amino and carboxylate groups of PLS, respectively, and presenting the phosphate group in close proximity to the nucleophilic Asp11 $O_{\delta 1}$.

The “pre-organisation” of the PLS substrate in the enzyme’s active site thus allows the acid–base catalysis by Asp13.

**Figure 6:**
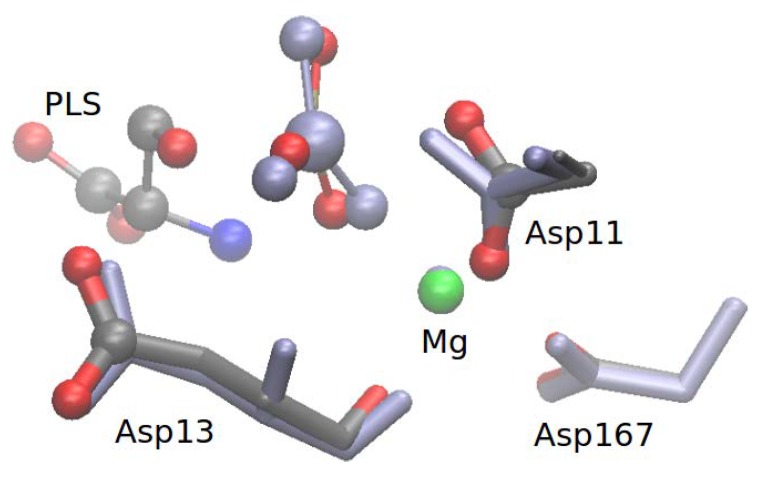
Superposition of the computed transition structure (coloured) with the crystal structure of the transition state analogue (1L7N) AlF${}_{3}$-PSP (blue). The phosphate adopts a trigonal planar configuration with the oxygen atoms of nucleophile (Asp11) and leaving group (PLS) at apical positions of a bipyramid. The P–O distance of the computed structure is ∼0.3 Å longer than the Al–O distance in the crystal.

Figure 6 represents a superposition of the transition structure with the serine bound transition state analogue from Ref. [26]. Both structures show a trigonal planar meta-phosphate (analogue). The P–O distances of 2.36 Å and 2.39 Å are significantly larger than the O–Al distance of 2.09 Å in the crystal structure. Whereas the smaller distance in the crystal structure might be interpreted as a stretched bond, the computed transition state geometry has a clear dissociative character.

#### 3.2.2. Reaction Path of Step 2: Hydrolysis of Phosphoryl-Aspartate 11

**Figure 7:**
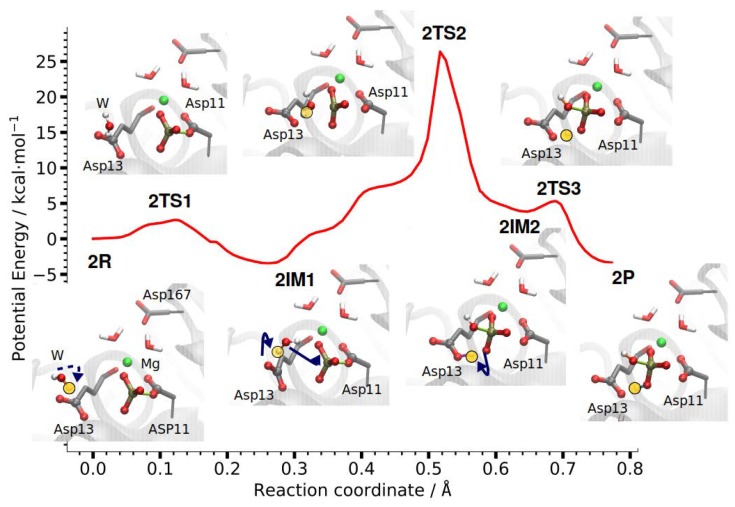
DFTB/MM energy profile of the second phosphate transfer step in PSP computed with CPR. The insets show active site residues of the reactant state, 2R, intermediate states 2IM1 and 2IM2, product state, 2P, and the transition states connecting them. The transferred proton is highlighted in yellow. Arrows indicate the transitions: The nucleophilic water molecule approaches the phosphate group and orientates for attack. The dissociation of the phosphoryl-aspartate and nucleophilic attack take place concertedly through a dissociative transition state, 2TS2. Upon the nucleophilic attack, a proton is transferred to Asp13 from the nucleophilic water molecule. The final step is the transfer of this proton from Asp13 to the phosphate group.

The second phosphate transfer step in PSP is the hydrolysis of the phosphoryl-aspartate intermediate (Figure 7). As can already be seen in the crystal structures, a water molecule is positioned in-line for nucleophilic attack on the phosphorous atom [26]. The attacking water molecule is found close to the former position of the substrate PLS in Step 1, with the water-oxygen to phosphorus distance being 5.3 Å. The hydrolysis reaction is initiated by the water molecule further approaching the phosphate group and rotating such that it is hydrogen-bonded to the phosphate group and Asp13.

Then, a proton is transferred from the nucleophilic water molecule to the carboxyl group of Asp13 and the thus-generated hydroxide ion attacks the phosphorus atom while the P–O bond to Asp11 breaks. Finally, the proton from Asp13 is transferred to the newly formed hydrogen-phosphate ion (Figure 7). The barrier (the energy of the highest transition state) for this second phosphate transfer step in PSP is 26.3 kcal/mol and the reaction energy for Step 2 is −3.4 kcal/mol.

The second step is thus significantly less catalysed by the enzyme, compared to the first step, though pre-orientation of all players, the attacking water molecule, the phosphorylated Asp11 and the general base Asp13, is also present in Step 2. Still, the barrier for this second phosphate transfer reaction in phosphoserine phosphatase is considerably lower than that of an uncatalysed phosphate hydrolysis [11,34].

#### 3.2.3. Reaction Coordinates

**Figure 8:**
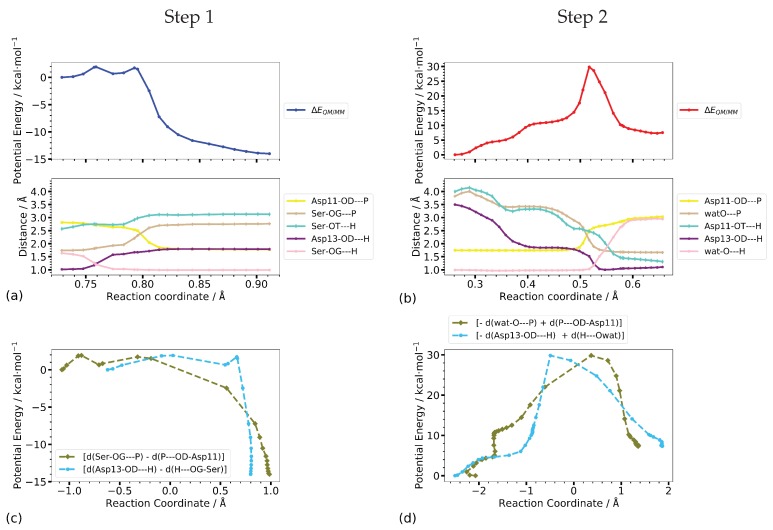
QM/MM minimum energy reaction path profile computed with CPR (top) and important distances (bottom) along the canonical reaction coordinate for the phosphate transfer in (**a**) Step 1 and (**b**) Step 2 of the dephosphorylation reaction in phosphoserine phosphatase. (**c**,**d**) QM/MM energy as function of the combined reaction coordinates for P–O bond breaking and formation and proton transfer in Step 1 and Step 2, respectively. Energies (in kcal·mol${}^{-1}$) are relative to the intermediate state from which the actual phosphate transfer starts (1IM2 in Figure 5 for Step 1 and 2IM1 in Figure 7 for Step 2, respectively).

Step 1 of the phosphate transfer reaction is first the rotation of and a subsequent proton transfer from the phosphorous OH-group to Asp13 (transitions 1R to 1IM1, and 1IM1 to 1IM2, respectively) as is well reflected in the corresponding O–H distances (Ser-OT–H and Asp13-OD–H in Figure 8a). The actual phosphate transfer, i.e., the transition from intermediate 1IM2 to product, commences at a value of the canonical reaction coordinate of RC = 0.54 Å. Close to the two transition states, 1TS3 and 1TS4, the O–H distances Ser-OG–H and Asp13-OD–H, and Ser-OG–P and Asp11-OD–P, respectively, are almost equal (Figure 8a,c)

For Step 2, the initial part of the mechanism is dominated by the nucleophilic water molecule approaching the phosphate group and orientating in-line for subsequent attack. Accordingly, the P–O-wat distance (and to some extent also the Asp13-OD–H-wat distance) describe the translation of the water molecule rather well, but must fail for its rotation. Regarding the actual phosphate transfer, starting at the canonical reaction coordinate value of RC = 0.3 Å, the combination of the two distances between the P atom and the O atom of the leaving group and nucleophile, respectively, provides a reaction energy profile almost identical to the one computed along the canonical reaction coordinate. In addition, the energy as a function of the combined distances between the transferred proton and the accepting and donating oxygen atoms shows a shape very similar to the reaction profile of the full coordinate set (Figure 8b,d), respectively). Only the final transition of the proton from Asp13 to the phosphate group is not properly described by the combination of the distances Asp13-OD–H and H–O-wat.

The individual distances (shown also in Figure 8b) reveal that the rise in energy follows the decreasing nucleophile—phosphorous distance. The rate-determining, i.e., energetically highest, transition state of Step 2 is located at RC = 0.53 Å after the two P–O distances are about equal and at about the crossing point of the O–H distances (RC = 0.54 Å), corresponding to a value of zero for their negative linear combination. This suggests already that the P–O distances form an acceptable reaction coordinate, with some improvement by inclusion of the O–H distances.

### 3.3. Free Energy Calculations

#### 3.3.1. One-Dimensional Reaction Profiles

To assess the entropic contribution of the protein environment to the catalytic reaction, we performed free energy calculations using umbrella sampling along different reaction coordinates. The projection of the minimum energy pathways, computed with CPR, shows that a combination of the P–O distances of the forming and breaking bonds as well as distances of the transferred proton to its donor and acceptor oxygen atoms largely reproduces the reaction energy profile of the phosphate transfer steps.

**Figure 9:**
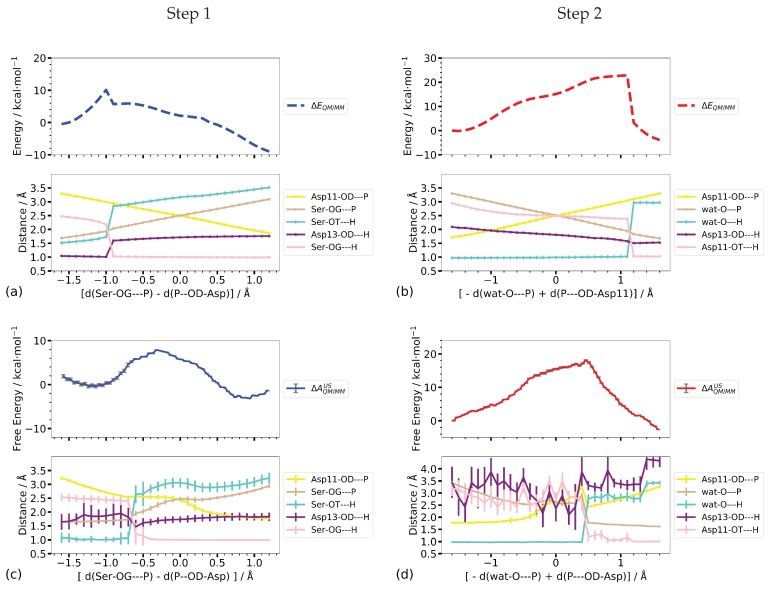
Reaction profiles from one-dimensional (**a**,**b**) potential energy scan and (**c**,**d**) umbrella sampling simulations, together with the evolution of important distances. The error bars for the distances from the umbrella sampling simulations represent the fluctuation of the distance in the respective window.

Figure 9 shows the computed potential energy profiles along the one-dimensional scans and the umbrella sampling calculations, respectively, along the same reaction coordinates. For both steps, the profiles from the potential energy scan of the combined P–O distances ([d(SerOG–P) − d(P–OD-Asp11)] and [−d(watO–P) + d(P–OD-Asp11)], respectively) are not smooth but rather show a sudden change in potential energy which coincides with the proton transfer not included in the reaction coordinate (see Figure 9c).

When employing umbrella sampling along the same reaction coordinate the transition free energy and the reaction energy of Step 1 are significantly higher (8.2 ±0.1 and 18.1 ± 0.2 kcal/mol, respectively) than in the scanned reaction profiles (1.6 and −14.3 kcal/mol, respectively, see Table 1). Looking at the distances along the free energy pathway, one notices that the proton is located at the phosphate group and not at Asp13, representing a state that corresponds to 1IM1 rather than to 1IM2. State 1IM1 is 7 kcal/mol lower in potential energy than 1IM2, explaining the spontaneous transition of the proton that occurs already during the equilibration phase of the umbrella sampling simulation. Note that this “proton back transfer” is not observed in the potential energy scans. The free energy barrier of 8.2 ±0.1 kcal/mol, computed by one-dimensional umbrella sampling along the combined P–O distances, corresponds to the transition from 1IM1 to product, which, on the CPR-computed pathway has a rather similar barrier of 8.6 kcal/mol. However, the reaction energy of −2.0±0.0 kcal/mol, for this step, is significantly lower than the −7.3 kcal/mol potential energy difference between the fully optimised states, 1IM1 and 1P, despite the similarity in the coordinates of the product states.

Regarding Step 2, the transition and reaction energies computed by the potential energy scan (22.8 and −3.9 kcal/mol, respectively) are rather close to the values obtained from umbrella sampling (18.1 ± 0.2 and −2.6 ± 0.0 kcal/mol, respectively). For Step 1, the transition state along the free energy profile is “later”, i.e., at a higher value of the reaction coordinate, than the sharp kink in the potential energy profile, associated with the proton transfer. Comparison of the P–O and O–H distances (listed in Table 2) of the transition states with those from the CPR-computed profile shows that in the latter the proton transfer from Asp13 to Ser-OG has not yet occurred. This is not reproduced in either the potential energy scan profile or the free energy profile, in both of which the proton has already been transferred to Asp13. Moreover, the Ser-OG–P distances at the transition states along both profiles, from the scan and from umbrella sampling, are longer than in the CPR-computed transition state, corresponding to an already completely broken bond.

In contrast, the proton transfer of Step 2 occurs “later” along the potential energy profile than along the free energy profile, that is before and after the proton transfer from the attacking water molecule to the phosphate group, respectively. In both cases, the new P–O-wat bond is already formed (see Figure 9) whereas in the reaction pathway computed with CPR, the transition state is truly dissociative with both P–O bond lengths >2 Å. The distance between Asp13 and the transferred proton fluctuates considerably in the umbrella sampling simulations due to Asp13 moving freely and unrestrained by the bias potential along the reaction coordinate.

Despite the comparable energy barriers in the profiles along the linear combination of the two P–O distances for Step 1 and Step 2, the different locations of the transition states, as manifested by P–O and O–H distances, show that this one-dimensional coordinate is not a sufficient projection of the actual reaction coordinate for the phosphate transfer.

#### 3.3.2. Two-Dimensional Reaction Profiles

**Figure 10:**
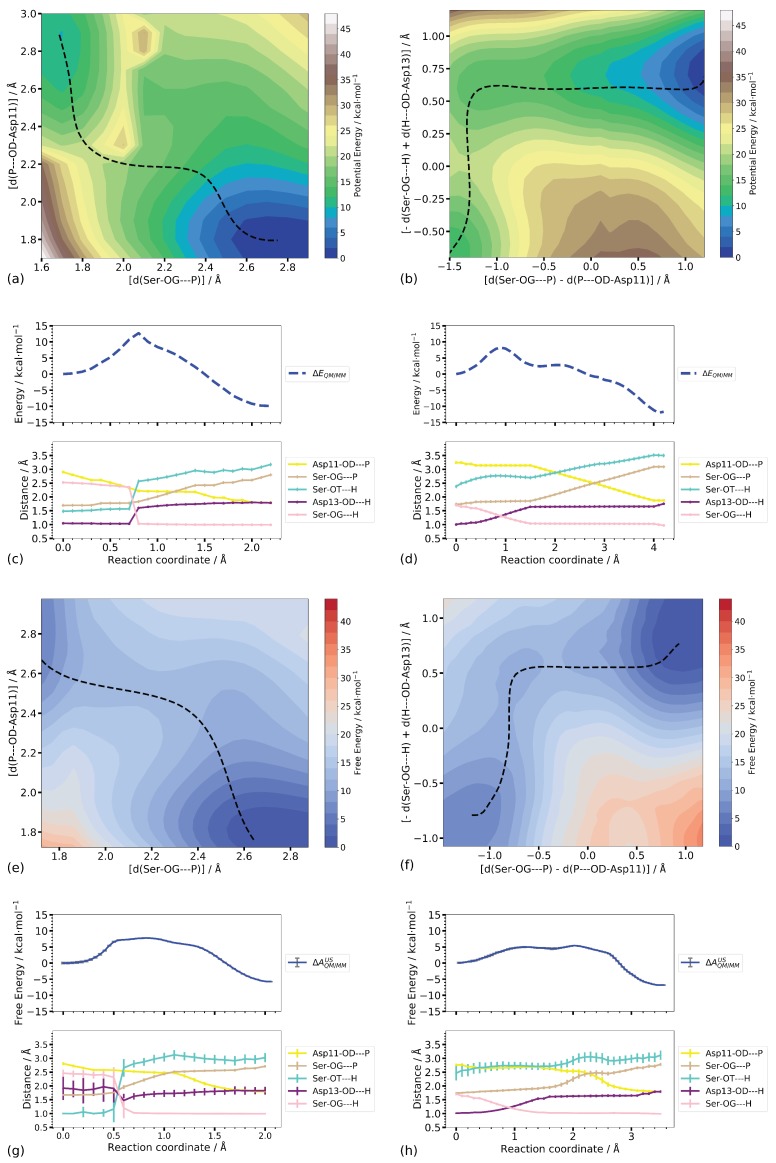
(**a**,**b**) Two-dimensional potential energy surfaces of Step 1. The dashed lines indicate the minimum energy pathway. (**c**,**d**) Energy profiles of the minimum energy paths in Step 1 (top) together with important distances (bottom). The reaction coordinate is the accumulated path lengths. (**e**,**f**) Two-dimensional free energy surfaces from umbrella sampling. The dashed lines indicate the minimum free energy pathways. (**g**,**h**) Free energy profile (top) together with important distances (bottom). The reaction coordinate is the accumulated path length.

**Figure 11:**
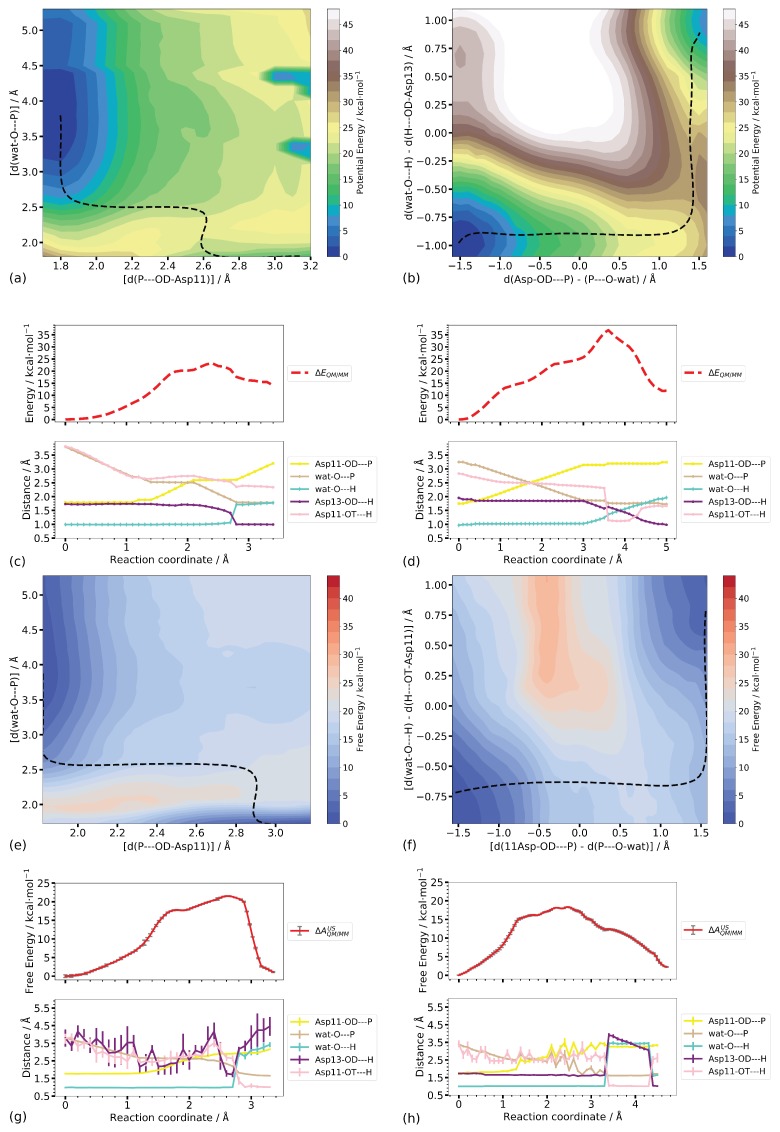
(**a**,**b**) Two-dimensional potential energy surfaces of Step 2. The dashed lines indicate the minimum energy pathway. (**c**,**d**) Energy profiles of the minimum energy paths in Step 2 (top) together with important distances (bottom). The reaction coordinate is the accumulated path lengths. (**e**,**f**) Two-dimensional free energy surfaces from umbrella sampling. The dashed lines indicate the minimum free energy pathways. (**g**,**h**) Free energy profile (top) together with important distances (bottom). The reaction coordinate is the accumulated path lengths.

To see the whether the one-dimensional reaction coordinate can be improved by considering the two P–O distances individually, or by including the linear combination of the O–H–O distances, we performed potential energy scans and umbrella sampling simulations in the respective two dimensions, that is using in one case the two P–O distances as reaction coordinates, and in the other case the linear combination of the P–O distances and a linear combination of the O–H distances as reaction coordinates. The resulting potential energy landscapes and the free energy landscapes from the umbrella sampling simulations are shown in Figure 10 for Step 1 and Figure 11 for Step 2, respectively.

#### Step 1

The minimum energy pathways of Step 1 on the potential and free energy surfaces computed along the two P–O distances both pass a transition state in which the proton transfer from Asp13 to the leaving group Ser-OG has already occurred (see Figure 10c,g), in contrast to the transition state of the CPR-computed reaction pathway. In the transition state obtained from the umbrella sampling simulations, the SerOG–P distance is, moreover, longer than in the transition states on the potential energy landscapes. The computed barriers are similar to those obtained from the one-dimensional profiles for both, the scan and umbrella sampling calculations (≤2 kcal/mol difference, see Table 1).

Employing the combined O–H distances as a second dimension instead of considering the two P–O distances individually (that is regarding them as a combined coordinate) results in lower transition barriers on both, the potential and free energy landscapes (Figure 10). In both cases, the location of the transition state shows an almost intact Ser-OG bond, albeit somewhat elongated in the case of the free energy simulation. According to the O–H distances, the proton is already transferred from Asp13 to the leaving group oxygen atom, reaching a state corresponding to 1IM3 on the CPR-computed pathway. The free energy profile does not suggest this intermediate to be stable, whereas a shoulder in the scanned profile suggests some metastability (Figure 10h,d, respectively). The formation of a transient meta-phosphate, at about equal P–O distances, which is the second transition to the product in the CPR-computed pathway, is not clearly visible as an individual barrier in either the scanned profile or the free energy profile (Figure 10d,h).

Potential energy and free energy barriers (8.2 and 5.4 ± 0.1 kcal/mol, respectively) are somewhat lower than in the two-dimensional profiles computed without inclusion of the O–H distances as a reaction coordinate, but still significantly higher than the CPR-computed barrier for the phosphate transfer in Step 1.

The reaction energies on either of the potential energy landscapes (−9.9 and 12.0, respectively) are closer to the CPR-computed reaction energy (−14.3 kcal/mol) than those computed on the free energy surfaces (−5.7± 0.1 and −6.8 ± 0.5 kcal/mol, respectively). Comparison of the P–O and O–H distances (Table 1) shows a high similarity of the product states on the potential and free energy surface spanned by the P–O distances with the fully optimised product state. In the product state on the potential energy surface spanned by the linear combinations of P–O and O–H distances, the protonated leaving group Serine is farther from Asp11 and Asp13 than in the other product states, as manifested by the larger P–OG–Ser and Ser–OT–H distances (Table 2). The product state on the corresponding free energy surface (Figure 10f), however, exhibits distances similar to the product states computed for the other two surfaces and by CPR. This indicates that the conformation of the phosphate transfer end state is reasonably well reproduced by both two-dimensional representations, and the difference between relative free and potential energies can be attributed to an entropic effect.

Compared to the potential energy barrier for the phosphate transfer n Step 1, computed by CPR (1.6 kcal/mol), all transition state energies, from the scan and from the umbrella sampling simulations, are substantially higher. In the case of the umbrella sampling simulations along the two P–O distances, the starting point is, however, not a (meta-)stable state with the proton at Asp13, but rather a spontaneous proton transfer back to the phosphate group, forming a 1IM1-like state, which has occurred already in the equilibration phase of the corresponding simulation. This is similar to the situation in the one-dimensional umbrella sampling simulations. Again, this “proton back transfer” is not observed in the potential energy scans. The computed free energy barrier of 7.8 ± 0.1 kcal/mol is almost the same as the potential energy difference (8.6 kcal/mol) between 1IM1 and 1TS3, computed by CPR. Accordingly, the reaction energy of −2.8 kcal/mol along the CPR pathway is actually −7.3 kcal/mol, when considering the transition from 1IM1 to product. This is still less than the CPR-computed reaction energy, but significantly closer. When the O–H distances are used as additional coordinates, the proton is biased to stay at Asp13 such that these simulations represent the transition from 1IM2 to the product state. It is interesting to note that only the two-dimensional scan along the combined P–O and combined O–H distances reproduces a transition state with the proton located “between” Asp13 and the leaving group, as is observed on the CPR-computed pathway. In the case of the umbrella sampling simulations along those coordinates, however, a region of higher energy that can be considered a transition region, includes the proton transition with equal O–H distances in its beginning as well as equal P–O distances towards its end.

#### Step 2

Using the two P–O distances d(P–O-Asp11) and d(wat-O–P) as reaction coordinates, both the minimum energy pathways computed on the potential energy surface as well as that computed on the free energy surface show shoulders in their energy profiles before the actual highest energy transition state. The location of the shoulder corresponds to about equally long P–O distances of ∼2.5 Å, representing a meta-phosphate in a dissociative mechanism. The highest point on the energy profiles, however, is found for the transition of a proton from the attacking water molecule (wat-O), to Asp13 in the case of the potential energy scan, and to the phosphate group (Asp11-OT) in the free energy calculations. The corresponding distances are not contained in the reaction coordinate, that is, the proton transfer takes place spontaneously to a more favourable position that is different for the two types of calculation. Nevertheless, the barriers for this phosphate transfer step are similar on the potential energy and the free energy surface (23.3 and 21.5 ± 0.1 kcal/mol, respectively). The reaction energies, however, differ (14.1 and 1.1 ± 0.1 kcal/mol), in agreement with the different end states (proton at Asp13 and proton at the phosphate group, respectively) reached.

When employing the linear combination of P–O distances and O–H distances [d(wat-O–H) − d(H–OD-Asp13)] for the phosphate transfer step, on the potential energy pathway and on the free energy pathway, first a proton transfer from the attacking water nucleophile to the phosphate group is observed before, finally, the (forced) transition to Asp13 takes place (see Figure 11d,h). On the potential energy landscape, the first proton transfer to the phosphate group corresponds to the transition state (with a relative energy of 36.9 kcal/mol), whereas on the free energy surface this proton transfer occurs on a “downhill” part of the energy profile. The transition state on this profile is characterised by the elongated two P–O distances (∼2.6 Å) (see Table 3) and a significantly lower barrier 18.4 ± 0.1 kcal/mol) than on the potential energy surface. Though the end states, in the sense of having comparable P–O and O–H distances, are very similar, the relative potential and free energies differ (11.9 and 2.30 ± 0.1, respectively) by about the same (∼10 kcal/mol) as those for the end states computed using only the P–O distances as reaction coordinates. This suggests the entropic contribution contained in the free energies to have a larger effect than the (potential) energy difference caused by the different proton positions.

## 4. Discussion

### 4.1. Phosphoryl Transfer Mechanism and the Nature of the Transition States

Solution studies on the uncatalysed hydrolysis of the acetylphosphate dianion have led to the proposal of a dissociative mechanism with a meta-phosphate species that is subsequently captured by water [74]. In contrast, it has been suggested that the interaction with the enzymatic cavity might retard meta-phosphate formation [25], and that activation of the water nucleophile by a general base (as found in the present calculations) might affect the mechanism by replacing the dissociative hydrolysis pathway seen in solution by an associative mechanism in the enzyme [75]. Indeed, a proposed mechanism for the PSP reaction based on the distances and structure of the transition-state-analogue complex of phosphoserine phosphatase-AlF${}_{3}$ invokes an associative pathway [26]. However, as stated in Ref. [26], the real transition state might have a geometry and charge distribution very different from the analogue. In the QM/MM study by Re at al. [53], a dissociative pathway is found for Step 1 of the phosphoryl transfer mechanism and also, in our present computations, the mechanisms of both Step 1 and Step 2, are clearly dissociative: As also observed in the previous study on Step 1 [53], the meta-phosphate and oxygen atoms of the nucleophile and the leaving group are in apical positions resembling a trigonal-bipyramidal associative transition state, similar to that mimicked in the crystal structures of the PSP-BeF${}_{3}$ and PSP-AlF${}_{3}$ complexes [26]. However, in the present optimised transition structures and the transition states on the free energy landscapes, the P–O distances are significantly longer than observed in the transition state analogues, clearly consistent with a dissociative character of the reaction mechanism.

The potential energy barrier of the phosphate transfer in Step 1, computed in the present work is 1.6 kcal/mol, using CPR. This is lower than the 4.0 kcal/mol computed by Re et al. [53] but still in good agreement given the different approaches used to obtain the minimum energy pathway, i.e., potential energy scan and CPR, as well as the different QM methods employed (semi-empirical DFTB in this work and DFT in [53]. According to our active-site model calculations, the energy barrier for the P–O bond dissociation and the associated proton transfer (see Table S1, TS11) is excellently reproduced by the semi-empirical approach. The second barrier, corresponding to the aspartyl-phosphate formation, and the energy of the product state are, however, underestimated in DFTB. Using also a two-dimensional scan, but with a slightly different proton transfer coordinate, we find a barrier of 8.2 kcal/mol, i.e., about as much higher as the CPR-computed barrier is lower than the values reported in the previous study employing density functional theory (B3LYP/MM) [53]. These not too different barriers and a similar mechanism, obtained by a different path finding method, on the one hand confirm the previous results and on the other hand render the semi-empirical treatment with DFTB suitable for the exploration of the phosphate transfer reactions in PSP and possibly other, similar enzymes.

### 4.2. Choice of Reaction Coordinates

The projection of the CPR-computed pathways, i.e., along a canonical reaction coordinate, onto the P–O and O–H distances shows these to change rather smoothly along the mechanism. The energy profile as a function of linear combinations of P–O and O–H distances suggests these as a good projection of the canonical reaction coordinate onto lower dimensions. The sharp kink observed in the reaction profiles computed from one-dimensional potential energy scans indicates that the linear combination of the P–O distances is insufficient, in agreement with the previous study in ref [53], likely due to “corner cutting” by the spontaneous transition of a proton from Asp13 to the leaving group. A spontaneous transition, not contained in a reaction coordinate, is also observed in the umbrella sampling simulations. For Step 1, this even leads to a different starting point. Treating the two P–O distances as two separate coordinates does not remedy this behaviour. The free energy profiles, one-dimensional or along the two P–O distances, are rather smooth, compared to the potential energy scans. However, the clear under-sampling of the proton transfer (observed only once as a consequence of proceeding along the P–O distances) leaves an intrinsic error in the corresponding free energies. Since the conformations with the proton at the other, not observed position, are likely comparably high in energy, their contribution to the relative free energy of a particular state (as defined by the P–O distances) may be only small.

Inclusion of the O–H distances as reaction coordinates, and thus better, though biased, sampling of proton transfer states, does not change the order of events. That is, the proton transfer takes place before P–O bond breaking and formation in the phosphate transfer reaction of Step 1 and the opposite, i.e., first P–O bond breaking and formation and then proton transfer, is observed for Step 2. This, together with an elevated energy at the biased and unbiased proton transfer, indicates that the proton transfer does not occur “too early”, thus not changing the mechanism compared to the unbiased CPR calculation or, at least qualitatively the reaction energy profile. The free energy barriers computed from two-dimensional sampling along the P–O and O–H distances are lower than those obtained when using only the P–O distances (as one or two dimensions), for both Step 1 and Step 2.

Interestingly, the one-dimensional energy scans of both phosphate transfer steps show higher barriers (by 2 and 0.5 kcal/mol, respectively) than their two-dimensional counterparts using only the O–P–O distances. For Step 2, the two-dimensional scan using the combined P–O and O–H distances as reaction coordinates results in a significantly (∼14 kcal/mol) higher barrier. This, together with the kinks observed in the corresponding 1D-energy profiles suggests the system has “escaped” along other degrees of freedom.In the umbrella sampling simulations, the two-dimensional treatment results in lower free energy barriers for Step 1 using either set of reaction coordinates. For Step 2, the free energy barrier is actually higher if using only the P–O distances and about the same if the proton transfer coordinate is included. A lowering of free energy barriers by only increasing the dimensionality, i.e., using the P–O distances separately, is only observed for Step 1, and this is supposedly largely due to the different starting points with respect to the proton transfer coordinate (proton at Ser-OT and not at Asp13). The improved treatment of the proton transfer coordinate, in contrast, results in reduced free energy barriers in both steps.

However, for Step 2, using the combined wat-O–H and H–OD-Asp13 distances as the reaction coordinate seems to be still insufficient, as evidenced by the direct proton transfer to the phosphate group (OT-Asp11) without intermediate binding to Asp13 (as in the CPR-computed reaction mechanism). This event, however, takes place after the actual transition state, i.e., the highest energy point along the path, has been passed and therefore has little impact on the computed free energy barrier. Such a case could be addressed with higher-dimensional sampling techniques, such as multi-dimensional umbrella sampling, meta-dynamics using several collective variables [76,77,78], its variant transition-tempered meta-dynamics [79], or the string method combined with Hamiltonian replica exchange simulations [80,81], which, in a QM/MM framework, are all computationally very expensive. From the present resemblance of the mechanism and most free energy barriers computed for the phosphate transfer reaction in PSP, however, we are confident that the two-dimensional setup in this work is sufficient, at least when the most relevant distances, as obtained from the CPR-computed pathways, are contained in the reaction coordinates.

### 4.3. Enzymatic Efficiency of PSP

The barriers of the uncatalysed reaction in water—found for both associative and dissociative mechanisms—typically range within ∼35–40 kcal/mol [11,34]. This is in good agreement with the barriers for the uncatalysed reaction found in the present small model calculations (Tables S1 and S2). The rate of acetylphosphate hydrolysis under physiological conditions [74] is also very low (∼$10^{-4}$ s${}^{-1}$). In contrast, aspartylphosphate hydrolysis catalysed by HAD superfamily members is in general at least 10${}^{5}$ times faster than in solution [6]. Consistent with this, the catalytic cycle of PSP found in this study has a rate limiting free energy barrier of ∼18–22 kcal/mol (depending on the reaction coordinates used) for dissociation of the phosphoryl-aspartate in Step 2, which is in very good agreement with the barrier of ∼21 kcal/mol calculated according to transition state theory using the experimental k${}_{\mathrm{cat},PSP}$ = 20 min${}^{-1}$ measured at ∼$70{\;}^{\circ }$C [26]. The experimental and computational barriers found in other phosphohydrolases are also in very good agreement with the present work [82,83].

The efficiency of the catalytic mechanism herein proposed is thus clearly shown, although the actual turnover of PSP may well be affected by effects not studied here, such as PLS substrate binding, the release of l-serine and P${}_{i}$, or the conformational changes leading back to the apo-enzyme.

The fact that Step 1 has an almost negligible barrier compared to Step 2 suggests that this first part of the phosphoryl-transfer process has higher demands on the enzymatic catalyst. It has been proposed that in Step 2 there is less variation between HAD superfamily members in electron polarization of the aspartylphosphate moiety and activation of the water nucleophile by the enzyme [25]. Orientation and deprotonation of the water through a nearby base have also been suggested to play a crucial role, enhancing the rate of dephosphorylation by typically 10${}^{2}$∓10${}^{4}$ [84]. The present computational study finds the rate limiting barrier for the enzymatic activity of PSP is indeed the orientation and deprotonation of the nucleophilic water through Asp13 (and simultaneous P–O bond dissociation) in Step 2. Asp13’s catalytic function is its role as a general base in Step 2, whereas it acts as general acid in Step 1. For this to work, both the phospho-l-serine in the first step and the phosphoryl-aspartate in the second step must be properly positioned. The stabilisation of a catalytically competent conformation is provided by the other active-site residues, i.e., Glu20 and Ser99, Lys144, Asn170, Asp171, and essentially the Mg${}^{2+}$ cofactor. The crucial role of this environment is supported by the findings of mutation studies on human PSP [85].

For Step 1, the computed free energy barriers are higher than the potential energy barrier obtained from CPR. This indicates the transition state to be entropically unfavourable. This is in agreement with the delicately positioned players in the rate determining step: PLS, Asp13 and Asp11 must be precisely orientated in order for the choreography of proton transfer to the leaving group and subsequently the P–O bond dissociation and formation to work. Step 2, in contrast, shows favourable entropic contributions, as manifested by the lower free energy barriers compared to the potential energy values. The demands to place nucleophile (water), Asp13 and the phosphate group properly are as high as in Step 1 to ensure P-O bond dissociation, formation, and proton transfer to take place. Positioning of the water molecule is, however, achieved by Asp13 already, leaving residues such as Glu20 or Arg56 more conformational freedom in Step 2 of the phosphoryl-transfer in PSP. These findings suggest that variations of the active site structure, as found within the HAD superfamily, have little impact on the catalytic efficiency of the rate-limiting Step 2, as long as the Mg${}^{2+}$-ion and the two essential aspartate residues (here Asp11 and Asp13) are unaltered. The further composition of the active site may, however, be responsible for specificity in substrate binding and catalysis.

## 5. Conclusions

We have presented a computational investigation of the phosphatase activity of PSP, a characteristic member of the HAD I superfamily of enzymes. Both Steps 1 and 2 show a distinct dissociative character presenting a trigonal-planar meta-phosphate transition state species.

The present computations agree with and extend a general mechanism suggested for most HAD phosphotransferases, which utilizes a nucleophile in Loop 1, followed two positions downstream by a general acid–base that binds and, in many cases protonates, the substrate-leaving group of Step 1 and, possibly, binds and deprotonates the nucleophile of Step 2 [25]. In the present specific case of PSP, proton transfer from the anionic substrate PLS to the general acid–base Asp13 and subsequent protonation of the substrate-leaving group accompanies and facilitates phosphoryl transfer in Step 1. Consistently, in Step 2, Asp13 acts as general base by deprotonating the nucleophilic water, coinciding with the capture of the resulting hydroxyl ion and followed by a proton transfer from the acid Asp13 to the leaving-group P${}_{i}$. The metal cofactor Mg${}^{2+}$ indirectly assists catalysis by structurally orienting residues comprising the catalytic scaffold, thus ensuring that the substrate, the intermediate phosphoryl acceptor Asp11 and the general acid-general base Asp13 are poised for the two phosphate transfer reactions to occur.

We used here the combination of minimum energy pathway computation using a chain-of-states optimiser, such as CPR, that does not require an a priori definition of a reaction coordinate to explore the reaction mechanism with subsequent free energy calculations along an approximation of the thus identified reaction coordinate. Projections of the canonical reaction coordinate onto important distances suggests two variants of a two-dimensional, and therefore approximative, reaction coordinate. Our free energy simulations along those coordinates confirm the importance of explicit inclusion of the proton transfer coordinate, in particular in Step 1. For Step 2, the reaction pathways are qualitatively similar for both sets of reaction coordinates. The free energy calculations reveal Step 1 to be entropically disfavourable, whereas Step 2 is entropically favourable. The differences can be explained by the conformational demands needed to properly position the substrate and the nucleophile, involving a different number of residues in the two steps.

## Figures and Tables

**Table 1:** Energies of transition (TS) and product (P) state along Step 1 and Step 2 of the phospho- hydrolysis reaction in PSP, computed with conjugate peak refinement (CPR), one- and two-dimensional potential energy scans, and by one- and two-dimensional umbrella sampling (US). Energies are relative to the intermediate state (IM) from which the phosphate transfer starts (1IM2 in the CPR-computed pathway for Step 1, Figure 5 and 2IM1 in the CPR-computed pathway of Step 2, Figure 7). OPO refers to the linear combination of P–O distances ([d(Ser-OG–P) − d(P–O-Asp11)] for Step 1 and [d(P–O-Asp11) − d(wat-O–P)], for Step 2), OHO to the linear combination of O–H distances ([−d(Ser-OG–H) + d(H–OD-Asp13)] in Step 1 and [d(wat-O–H) − d(H–OD-Asp13)] in Step 2).

	Step 1		Step 2	
	TS	Prod	TS	Prod
CPR	1.6	−14.3	29.8	0.1
1D-Scan, OPO	10.7	−9.5	22.8	−3.9
1D-US, OPO	8.2 ± 0.1	−2.0 ± 0.0	18.1 ± 0.2	−2.6 ± 0.0
2D-Scan, OP, PO	12.7	−9.9	23.3	14.1
2D-US, OP, PO	7.8 ± 0.1	−5.7 ± 0.1	21.5 ± 0.1	1.1 ± 0.1
2D-Scan, OPO, OHO	8.2	−12.0	36.9	11.9
2D-US, OPO, OHO	5.4 ± 0.1	−6.8 ± 0.5	18.4 ± 0.1	2.3 ± 0.1

**Table 2:** Coordinates of end states (IM and P) and transition state (TS) along Step 1 of the phospho- hydrolysis reaction in PSP, computed with conjugate peak refinement (CPR), one- and two-dimensional potential energy scans, and by one- and two-dimensional umbrella sampling (US). Coordinate values from umbrella sampling are averaged over the simulation with the centre of the biasing potential at the reaction coordinate (RC or RC1 and RC2) values of the respective state (IM, TS, or P) on the free energy surface and may therefore differ from these values. OPO refers to the linear combination of P–O distances, [d(Ser-OG–P) − d(P–O-Asp11)], OHO to the linear combination of O–H distances, [−d(Ser-OG–H) + d(H–OD-Asp13)].

		RC	Ser-OG–P	P–O-Asp11	H–OD-Asp13	Ser-OG–H	Ser-OT–H
CPR	IM	0.73	1.74	2.81	1.01	1.64	2.56
	TS	0.76	1.83	2.71	1.23	1.20	2.74
	P	0.91	2.76	1.78	1.79	0.99	3.13
1D Scan	IM	−1.5	1.72	3.25	1.03	2.44	1.54
OPO	TS	−0.9	2.04	2.94	1.60	1.02	2.84
	P	1.2	3.09	1.87	1.76	0.99	3.52
1D US	IM	−1.3	1.66 ± 0.03	2.78 ± 0.05	1.84 ± 0.25	2.46 ± 0.13	1.00 ± 0.03
OPO	TS	−0.3	2.36 ± 0.09	2.54 ± 0.08	1.71 ± 0.13	1.01 ± 0.03	3.02 ± 0.18
	P	1.0	2.71 ± 0.06	1.80 ± 0.04	1.83 ± 0.13	0.99 ± 0.02	3.03±0.18
2D-Scan	IM	1.7, 2.9	1.69	2.90	1.05	2.52	1.48
OP,	TS	1.8, 2.2	1.83	2.22	1.60	1.03	2.57
PO	P	2.8, 1.8	2.80	1.79	1.78	0.99	3.18
2D-US	IM	1.7, 2.7	1.68 ± 0.03	2.72 ± 0.05	1.89 ± 0.31	2.44 ± 0.12	1.00 ± 0.03
OP,	TS	2.2, 2.5	2.22 ± 0.06	2.50 ± 0.05	1.68 ± 0.11	1.01 ± 0.03	2.87 ± 0.14
PO	P	2.7, 1.8	2.71 ± 0.05	1.80 ± 0.04	1.83 ± 0.14	0.99 ± 0.03	3.03 ± 0.17
2D-Scan	IM	−1.5, −0.7	1.74	3.24	1.00	1.70	2.38
OPO,	TS	−1.3, 0.0	1.84	3.14	1.31	1.31	2.77
OHO	P	1.2, 0.7	3.09	1.87	1.75	0.97	3.50
2D-US	IM	1.1, −0.8	1.74 ± 0.04	2.76 ± 0.05	1.01 ± 0.03	1.71 ± 0.06	2.46 ± 0.26
OPO,	TS	−0.6, 0.3	1.94 ± 0.05	2.63 ± 0.06	1.54 ± 0.04	1.04 ± 0.02	2.72 ± 0.13
OHO	P	1.0, 0.8	2.78 ± 0.06	1.78 ± 0.04	1.79 ± 0.05	0.97 ± 0.02	3.11 ± 0.17

**Table 3:** Coordinates of end states (IM and P) and transition state (TS) along Step 2 of the phospho- hydrolysis reaction in PSP, computed with conjugate peak refinement (CPR), one- and two-dimensional potential energy scans, and by one- and two-dimensional umbrella sampling (US). Coordinate values from umbrella sampling are averaged over the simulation with the centre of the biasing potential at the reaction coordinate (RC or RC1 and RC2) values of the respective state (IM, TS, or P) on the free energy surface and may therefore differ from these values. OPO refers to the linear combination of P–O distances, [d(P–O-Asp11) − d(wat-O–P)], OHO to the linear combination of O–H distances, [d(wat-O–H) − d(H–OD-Asp13)].

		RC	P–O-Asp11	Wat-O–P	H–OD-Asp13	Wat-O–H	H–OT-Asp11
CPR	IM	0.26	1.74	3.82	3.50	0.99	4.00
	TS	0.52	2.51	2.14	1.52	1.03	2.47
	P	0.64	3.04	1.66	1.12	2.93	1.28
1D Scan	IM	−1.5	1.75	3.25	2.06	0.97	2.88
OPO	TS	1.1	3.04	1.95	1.57	1.02	2.38
	P	1.6	3.30	1.68	1.52	2.90	1.02
1D US	IM	−1.6	1.78 ± 0.05	3.40 ± 0.07	3.44 ± 0.64	0.98 ± 0.03	3.18 ± 0.31
OPO	TS	0.5	3.26 ± 0.10	2.92 ± 0.10	3.87 ± 0.61	0.97 ± 0.02	2.65 ± 0.38
	P	1.6	3.26 ± 0.06	1.61 ± 0.03	4.33 ± 0.26	3.43 ± 0.08	1.01 ± 0.03
2D-Scan	IM	1.8, 3.8	1.79	3.78	1.73	0.99	3.81
OP,	TS	2.6, 2.1	2.60	2.20	1.61	1.01	2.62
PO	P	3.2, 1.8	3.20	1.78	0.99	1.77	2.34
2D-US	IM	1.8, 3.9	1.76 ± 0.04	3.61 ± 0.05	4.17 ± 0.35	0.98 ± 0.03	3.33 ± 0.30
OP,	TS	2.9, 2.3	2.94 ± 0.06	1.82 ± 0.04	3.16 ± 0.13	2.96 ± 0.23	1.35 ± 0.21
PO	P	3.1, 1.7	3.15 ± 0.05	1.65 ± 0.03	4.58 ± 0.53	3.42 ± 0.14	1.01 ± 0.05
2D-Scan	IM	−1.5, −1.0	1.75	3.25	2.83	0.96	1.95
OPO,	TS	1.4, −0.3	3.20	1.76	1.15	1.35	1.62
OHO	P	1.5, 1.0	3.24	1.73	1.66	1.97	0.98
2D-US	IM	−1.6, −0.8	1.77 ± 0.04	3.40 ± 0.07	1.72 ± 0.05	1.00 ± 0.03	3.41 ± 0.20
OPO,	TS	-0.5, -0.6	2.85 ± 0.06	1.67 ± 0.04	1.62 ± 0.06	1.01 ± 0.03	3.19 ± 0.37
OHO	P	1.6, 0.8	3.29 ± 0.06	1.68 ± 0.04	1.00 ± 0.02	1.74 ± 0.05	2.43 ± 0.26

## Supplementary Materials

This supplementary materials following are available online.

## Abbreviations

The following abbreviations are used in this manuscript:

PSPI	Phosphoserine phosphatase
PLS	phospho-l-serine
CPR	Conjugate Peak Refinement
MD	molecular dynamics
QM/MM	quantum mechanical/molecular mechanical
HAD	haloacid dehalogenase-like hydrolase
DFT	Density functional theory
B3LYP	Becke’s three parameter hybrid function
DFTB3/OB3	Density functional tight-binding method with Third-Order Parametrization for Organic and
	Biological Systems (3OB)
SCC-DFTB	Self-consistent-charge density-functional tight-binding
SCF	self-consistend field
NPT	constant number of particles, N, pressure P, and temperature, T
NVT	constant number of particles, N, volume, V, and temperature, T
WHAM	Weighted Histogram Analyis Method
RC	Reaction coordinate
