# Supplementary material for: Quantum Mechanical/Molecular Mechanical Analysis of the Catalytic Mechanism of Phosphoserine Phosphatase
Source: Molecules. 2018 Dec 17;23(12):3342. doi: 10.3390/molecules23123342 (PMC6321591; doi:10.3390/molecules23123342)
Supplement: Supplementary file 1 [file molecules-23-03342-s001.pdf]

Supplementary Material for: Quantum Mechanical/Molecular Mechanical Analysis of the Catalytic Mechanism of Phosphoserine Phosphatase

Dieter Krachtus[1,a], Jeremy C. Smith[1,2] and Petra Imhof[3]

December 11, 2018

1 Computational Molecular Biophysics Group,
Interdisciplinary Center for Scientific Computing (IWR),
Im Neuenheimer Feld 368, 69120 Heidelberg, Germany

a Present address: genflux.de
Kraichgaustrass 22
69151 Kleingemuend, Germany

2 University of Tennessee / Oak Ridge National Laboratory,
Center for Molecular Biophysics
One Bethel Valley Road, P.O. Box 2008,
Oak Ridge, TN 37831-6255, USA

Freie Universität Berlin
Institute for Theoretical Physics
Arnimallee 14
14195 Berlin, Germany
Email: petra.imhof@fu-berlin.de

1 System setup and model evaluation

1.1 Molecular Dynamics Simulation of the reactant of Step 1 with differently charged substrate PLS

Figure 1 shows selected distances in the course of the MD simulation used to determine the conformation and the more probable protonation state of the reactant state of the first phosphoryl-transfer in PSP .

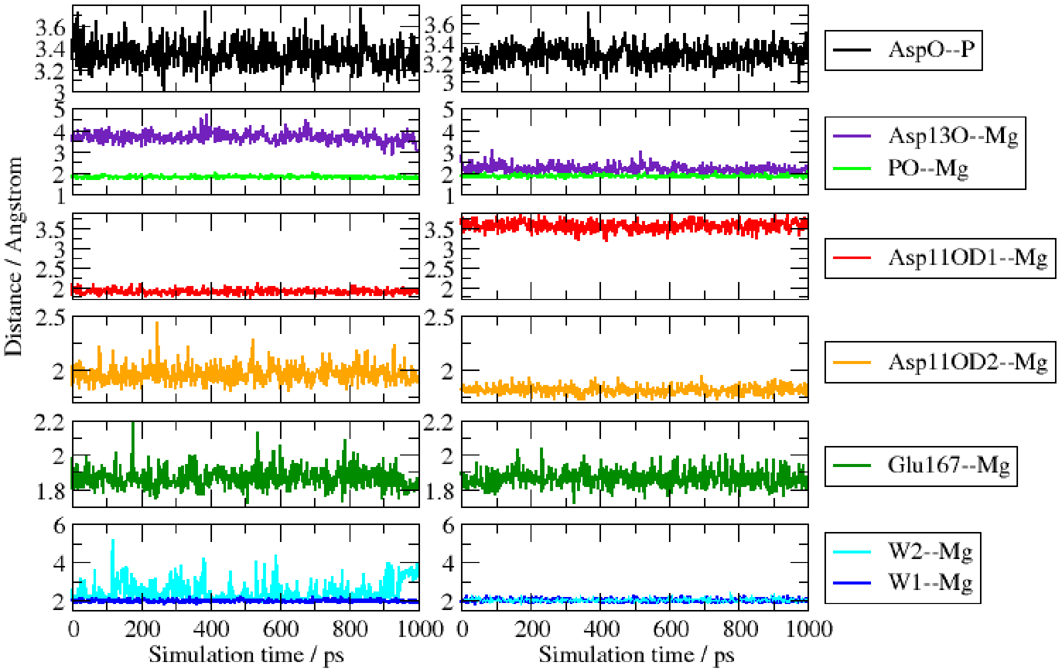

Figure 1: Distance of the ligand oxygen atoms to the central magnesium atom throughout the molecular dynamics simulations of dianionic (left) and anionic PLS (right). The carbonyl oxygen atom of Asp13 is replaced by the second carboxyl oxygen atom of Asp11 in simulations with dianionic PLS.

1.2 Active Site model calculations

Pathway calculations using a minimal model of the active site of PSP were performed to evaluate the semi-empirical method scc-DFTB against a density functional theory level calculations. The reaction in Step 1 proceeds via unaided auto-dephosphorylation (Figure 2): direct proton transfer from the substrate phosphate to the leaving-group oxygen atom upon dissociation and formation of a metaphosphate (R1-TS11-IM1), and is completed by Asp11 binding to the phophoryl-group (IM11-TS12-P1).

In the model for the second phosphate transfer step in PSP (Figure 3) the methanol moiety (mimicking the serine) in the product of the first step, P1, is replaced by a water molecule so as to constitute the reactant for the hydrolysis of the phosphoryl-aspartate, R2. First, the phosphate group dissociates from the phophoryl-Asp11 and again forms a metaphosphate (R2-TS21-IM2). The metaphosphate is then attacked by the nucleophilic water molecule which upon attack transfers one proton to the phosphate moiety (IM2-TS22-P2). In the inverse direction, Step 2 can be regarded as the Asp11 attacking a non-ester phosphate group.

The energies calculated for the stationary points of both steps are presented in Tables 1 and 2. For the first transition state and intermediate of Step 1, the energies calculated with DFTB agree well with those computed by density functional theory, whereas the second transition state and the product energy are underestimated. Single-point B3LYP/6-31++G(d,p) energies calculated at the DFTB-level, on the other hand agree much better with the full DFT results for the second transition state and product than for the first two stationary points. For the second step, the DFTB-calculated relative energies are in excellent agreement with the DFT-calculated values (off by 2 kcal/mol at most). This result deteriorates, however, for B3LYP/6-31++G(d,p) energies on the DFTB-optimised structures, indicating the the full DFTB computations provide a favourable compensation of errors in calculated geometries and corresponding energy values (Table 2).

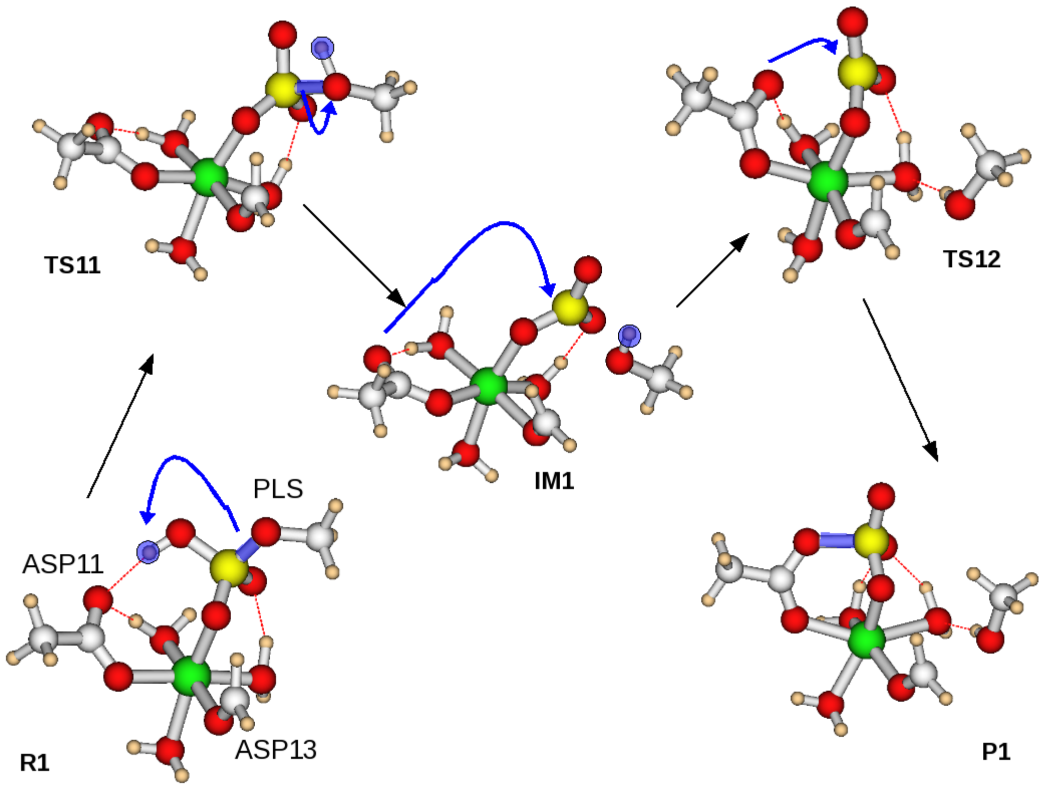

Figure 2: Stationary points along the first step of the phosphate transfer reaction in a minimal model of PSP. Only the chemical steps are shown, conformational transitions being omitted for clarity. P–O Bonds which are broken or formed and the proton being transferred are highlighted in blue

Table 1: Relative energies (in kcal/mol) of stationary points along the first step of the phosphate transfer reaction in a minimal model of PSP, calculated at different levels of theory

	B3LYP/ 6-31++G(d,p)	DFTB	B3LYP/ 6-31++G(d,p)// DFTB
R1	0	0	0
TS11	39	38	58
IM1	27	25	34
TS12	37	26	35
P1	30	18	27

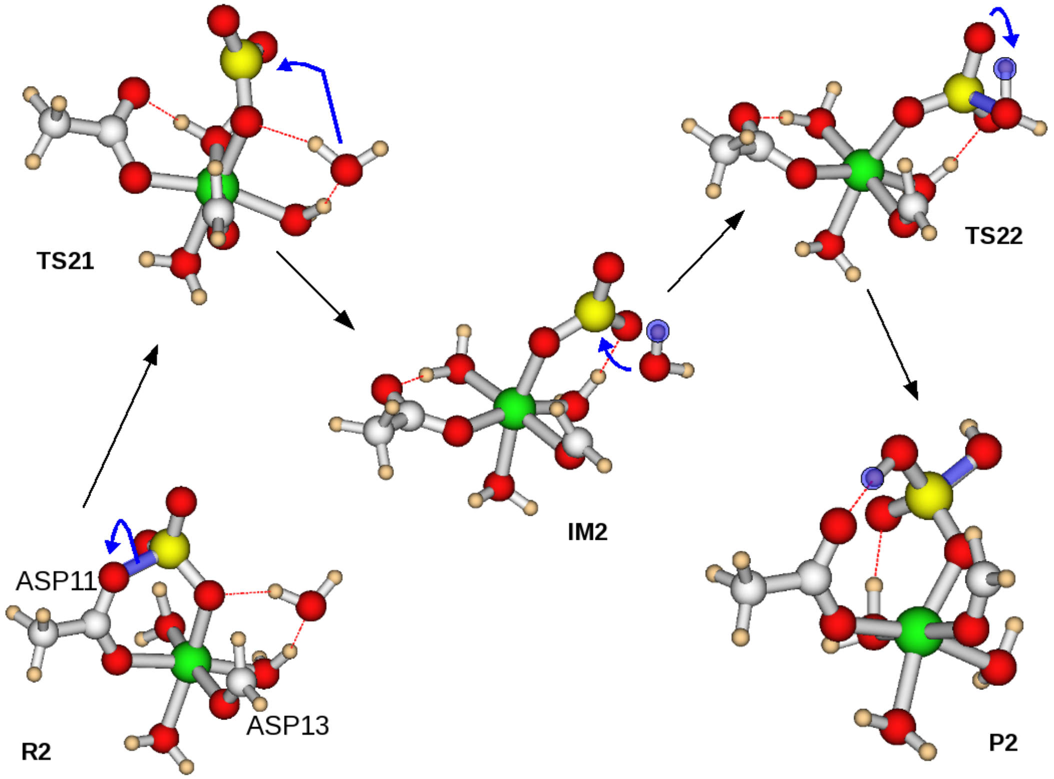

Figure 3: Stationary points along the second step of the phosphate transfer reaction in a minimal model of PSP. Only the chemical steps are shown, conformational transitions being omitted for clarity. P–O Bonds which are broken or formed and the proton being transferred are highlighted in blue

Table 2: Relative energies (in kcal/mol) of stationary points along the second step of the phosphate transfer reaction in a minimal model of PSP, calculated at different levels of theory

	B3LYP/ 6-31++G(d,p)	DFTB	B3LYP/ 6-31++G(d,p)// DFTB
R2	0	0	0
TS21	16	14	29
IM2	12	11	42
TS22	25	23	34
P2	-17	-16	-13

1.3 Size of the QM region

Validation of the size of the QM region was performed by calculating QM/MM pathways for Step 1 with all possible combinations of the differently-sized QM region and differently-sized region of flexible protein atoms. The resulting pathways were structurally and mechanistically very similar. The energy profiles (see Table 3) were reproducible, independent of the size of the QM region and the size of the flexible protein region. This further indicates that polarisation of the immediate vicinity of the smaller QM regions, which cannot be treated by the fixed-charge force field employed to describe the MM part of the system, has only little effect on the computed reaction pathway

Table 3: Impact of the size of the QM region on the computed QM/MM energies for the stationary points along the phosphate transfer pathways in phosphoserine phosphatase. The small region for Step~1 comprises the phospho-serine (PLS), the backbone of Asp13, sidechains of Asp11, Glu20, Ser99, Lys144, Asp167, the Mg ion, and two water molecule coordinating it (97 atoms). The large region (113 atoms) is extended by Asn170 and Asp171. For Step~2 the small QM region (86 atoms) consists of the phosphorylated Asp11, the backbone of Asp13, sidechains of Glu20, Ser99, Lys144, Asp167, the Mg ion, two water molecule coordinating it and the nucleophilic water molecule. The large region in steps 2 (102 atoms) also additionally includes Asn170 and Asp171 (See also Figure 1 in the main text).

	Step1		Step2	
	small	large	small	large
R	0	0	0	0
TS1	7.5	9.3	2.6	0.7
IM1	-2.8	-2.0	-3.5	-2.7
TS2	6.1	7.7	26.3	26.0
IM2	4.2	7.1	3.7	4.6
TS3	5.8	7.7	5.4	4.7
IM3	4.6	5.1		
TS4	5.6	5.9		
P	-10.1	-12.8	-3.4	-4.9

2 Coordinates of the B3LYP/6-31++G(d,p)-optimised stationary points of the minimal active site model

Table 4: B3LYP/6-31++G(d,p) optimized geometry of model R1

p	-3.5841	-0.7650	-4.4319
o	-4.4728	-0.4846	-3.2177
o	-2.5438	-1.8684	-4.1464
o	-2.8258	0.5395	-4.9820
mg	-1.7490	-1.9756	-2.2907
o	-1.3640	-4.1197	-2.7442
o	0.1764	-1.4358	-2.7475
o	-2.1113	0.0266	-1.6588
o	-3.6985	-2.3939	-1.5474
c	0.4572	-0.3834	-3.4276
o	-0.3425	0.5791	-3.5969
c	1.8524	-0.2888	-4.0176
h	2.5312	0.1001	-3.2486
h	1.8649	0.4018	-4.8629
h	2.2159	-1.2751	-4.3166
o	-0.7918	-2.5802	-0.4411
h	0.0679	-2.1573	-0.6074
h	-0.6229	-3.5328	-0.4249
h	-1.4854	0.4971	-2.2696
h	-3.0115	0.2772	-1.9427
h	-3.8974	-2.2004	-0.6226
h	-4.2153	-1.7380	-2.1087
c	-1.5594	-4.5878	-3.8565
h	-1.9684	-3.9575	-4.6599
h	-1.3270	-5.6479	-4.0539
o	-4.4728	-1.1228	-5.7314
c	-5.7030	-1.8459	-5.5768
h	-5.5129	-2.8798	-5.2636
h	-6.3509	-1.3541	-4.8458
h	-6.1793	-1.8488	-6.5592
h	-1.9028	0.5933	-4.6452

Table 5: B3LYP/6-31++G(d,p) optimized geometry of model TS11

p	-4.0383	-1.1270	-4.4742
o	-5.1224	-1.0553	-3.4209
o	-2.7526	-1.8271	-4.0422
o	-3.9256	-0.0457	-5.5756
mg	-2.1735	-2.3877	-2.1772
o	-2.0190	-4.4522	-3.0691
o	-0.1316	-2.3030	-2.2588
o	-2.1883	-0.5318	-1.2260
o	-4.1867	-2.7144	-1.6117
c	0.6395	-1.2721	-2.1682
o	0.2762	-0.1575	-1.7193
c	2.0709	-1.4496	-2.6412
h	2.6589	-0.5525	-2.4425
h	2.0697	-1.6558	-3.7173
h	2.5249	-2.3143	-2.1464
o	-1.6672	-3.5661	-0.3940
h	-0.7071	-3.4357	-0.4868
h	-1.8284	-4.5098	-0.5329
h	-1.2182	-0.2233	-1.3615
h	-2.7805	0.1790	-1.5002
h	-4.3839	-2.4916	-0.6931
h	-4.7201	-2.0987	-2.1992
c	-1.8914	-4.6439	-4.2679
h	-1.9392	-3.8064	-4.9791
h	-1.7331	-5.6669	-4.6495
o	-4.7231	-2.1331	-5.9038
c	-6.1012	-2.5636	-5.8888
h	-6.2093	-3.3477	-5.1362
h	-6.7664	-1.7309	-5.6434
h	-6.3327	-2.9622	-6.8793
h	-4.4810	-1.0444	-6.2734

Table 6: B3LYP/6-31++G(d,p) optimized geometry of model IM11

p	-4.3645	-0.7525	-4.0628
o	-5.1300	-1.1276	-2.8141
o	-2.8466	-0.7563	-3.9240
o	-5.0064	-0.2198	-5.2943
mg	-1.5917	-1.4627	-2.4751
o	-1.8676	-3.4566	-3.4291
o	0.2270	-1.4230	-3.4177
o	-1.2051	0.3742	-1.5721
o	-3.2189	-1.8870	-1.1893
c	1.0678	-0.4501	-3.4984
o	0.9993	0.6021	-2.8145
c	2.1946	-0.5961	-4.5051
h	3.0376	0.0429	-4.2353
h	1.8235	-0.2785	-5.4871
h	2.5106	-1.6390	-4.5897
o	-0.4680	-2.6556	-1.0431
h	0.3736	-2.7525	-1.5161
h	-0.8359	-3.5422	-0.9261
h	-0.2909	0.5965	-1.9894
h	-1.7960	1.1096	-1.7765
h	-3.2049	-1.4373	-0.3351
h	-4.0646	-1.6245	-1.6653
c	-1.8911	-3.5427	-4.6480
h	-1.7791	-2.6559	-5.2856
h	-2.0109	-4.5244	-5.1337
o	-4.5518	-2.9669	-5.1233
c	-5.5153	-3.8451	-4.5089
h	-5.0309	-4.2679	-3.6273
h	-6.4027	-3.2890	-4.1930
h	-5.7908	-4.6505	-5.1986
h	-4.9603	-2.5167	-5.8801

Table 7: B3LYP/6-31++G(d,p) optimized geometry of model TS12

p	-2.7244	0.0890	-4.1449
o	-3.3907	0.3579	-2.7995
o	-2.3292	-1.3863	-4.2881
o	-2.7861	1.0046	-5.3044
mg	-1.4249	-2.1399	-2.6075
o	-1.6164	-4.0976	-3.3380
o	0.3858	-1.5613	-3.3545
o	-1.1729	-0.4378	-1.1721
o	-3.5110	-2.1057	-1.8811
c	0.4473	-0.3672	-3.7909
o	-0.4243	0.5244	-3.4994
c	1.5899	0.0290	-4.6967
h	1.9830	1.0044	-4.3982
h	1.1962	0.1375	-5.7141
h	2.3780	-0.7260	-4.6933
o	-0.6760	-3.0922	-0.8522
h	-0.2343	-2.3860	-0.3553
h	-0.0597	-3.8301	-0.9500
h	-0.7627	0.1314	-1.8816
h	-2.0082	0.0268	-1.0059
h	-3.7403	-2.2140	-0.9490
h	-3.6828	-1.1441	-2.1473
c	-2.2165	-4.3763	-4.3776
h	-2.4476	-3.6027	-5.1188
h	-2.4467	-5.4239	-4.6114
o	-4.5428	-4.0739	-3.7636
c	-5.4772	-3.5575	-4.7150
h	-6.4138	-3.2597	-4.2268
h	-5.0660	-2.6987	-5.2608
h	-5.6954	-4.3615	-5.4222
h	-4.3348	-3.3778	-3.1127

Table 8: B3LYP/6-31++G(d,p) optimized geometry of model P1

p	-3.8915	-0.5502	-2.4221
o	-3.5079	0.0885	-1.0821
o	-3.1716	-1.9099	-2.6214
o	-5.2710	-0.4518	-2.9913
mg	-1.2440	-2.0221	-2.3139
o	-1.1663	-3.3757	-4.0438
o	-0.8685	-0.3131	-3.4226
o	-1.4001	-1.0470	-0.4467
o	-1.4803	-3.7063	-0.9948
c	-1.7013	0.5272	-3.8541
o	-2.9634	0.5321	-3.5943
c	-1.2396	1.6608	-4.7374
h	-1.1227	2.5562	-4.1154
h	-1.9939	1.8828	-5.4953
h	-0.2794	1.4243	-5.1991
o	0.8838	-2.1265	-2.3687
h	1.1252	-1.4207	-2.9897
h	1.2863	-2.9452	-2.6891
h	-0.7486	-0.4422	-0.0737
h	-2.3181	-0.5005	-0.6043
h	-1.5585	-3.1556	-0.1938
h	-2.3723	-4.0474	-1.1621
c	-2.1175	-3.5444	-4.8037
h	-3.0856	-3.0408	-4.6537
h	-1.9957	-4.2239	-5.6652
o	-5.1153	-2.2491	-5.1650
c	-6.2002	-2.1046	-6.0675
h	-6.0193	-2.7722	-6.9170
h	-7.1583	-2.3860	-5.6061
h	-6.2865	-1.0758	-6.4468
h	-5.2546	-1.6425	-4.4019

Table 9: B3LYP/6-31++G(d,p) optimized geometry of model R2

p	-3.6177	-1.4422	-3.5078
o	-3.3998	-0.8281	-2.1197
o	-2.5745	-2.5991	-3.7501
o	-4.9673	-1.6303	-4.0961
mg	-0.6919	-2.2869	-3.1549
o	-0.0330	-3.3521	-4.9967
o	-0.6884	-0.4437	-4.1317
o	-1.2024	-1.5769	-1.2748
o	-0.4532	-4.1603	-2.1956
c	-1.6438	0.1864	-4.6521
o	-2.8892	-0.1483	-4.5825
c	-1.3839	1.4548	-5.4274
h	-1.6993	2.3061	-4.8134
h	-1.9880	1.4705	-6.3379
h	-0.3233	1.5548	-5.6631
o	1.3895	-1.8106	-2.9838
h	1.5165	-1.0430	-3.5627
h	2.0144	-2.4943	-3.2611
h	-0.7224	-0.8773	-0.8174
h	-2.1816	-1.2123	-1.5104
h	-1.1690	-4.7624	-2.5288
h	-0.6775	-3.9524	-1.2757
c	-0.8123	-3.7594	-5.8471
h	-1.8893	-3.5548	-5.7646
h	-0.4339	-4.3373	-6.7065
o	-2.5679	-5.2125	-3.4685
h	-2.8634	-4.2699	-3.5935
h	-3.3365	-5.7286	-3.1981

Table 10: B3LYP/6-31++G(d,p) optimized geometry of model TS21

p	-3.9023	-1.8629	-3.2759
o	-3.9948	-1.6751	-1.7927
o	-2.6590	-2.6537	-3.7540
o	-4.9326	-1.5178	-4.2842
mg	-0.6928	-2.2148	-3.2441
o	-0.1679	-3.4402	-5.0135
o	-0.4452	-0.5640	-4.4181
o	-1.2735	-1.1675	-1.5172
o	-0.4001	-4.0043	-2.1460
c	-1.2712	0.4241	-4.3275
o	-2.1217	0.5260	-3.4093
c	-1.2148	1.4827	-5.4123
h	-0.2171	1.5450	-5.8537
h	-1.5264	2.4511	-5.0152
h	-1.9227	1.2028	-6.2021
o	1.4330	-1.9308	-3.0701
h	1.5437	-1.1995	-3.7006
h	2.0075	-2.6553	-3.3504
h	-1.5126	-0.3233	-1.9864
h	-2.1597	-1.4654	-1.2184
h	-0.4026	-3.9040	-1.1854
h	-1.0640	-4.6981	-2.3864
c	-0.8856	-3.5559	-5.9943
h	-1.9007	-3.1319	-6.0055
h	-0.5223	-4.0970	-6.8833
o	-2.3894	-5.3646	-3.3052
h	-2.7506	-4.4813	-3.5399
h	-3.1210	-5.9004	-2.9753

Table 11: B3LYP/6-31++G(d,p) optimized geometry of model IM2

p	-4.3172	-1.0946	-4.2915
o	-5.1089	-1.6107	-3.1133
o	-2.8208	-0.9346	-4.0639
o	-4.9362	-0.6607	-5.5717
mg	-1.5747	-1.5437	-2.5510
o	-1.6582	-3.5578	-3.4708
o	0.2669	-1.3591	-3.4154
o	-1.3965	0.3316	-1.6664
o	-3.2218	-2.1021	-1.3387
c	1.0252	-0.3198	-3.4744
o	0.8301	0.7350	-2.8187
c	2.2179	-0.3952	-4.4107
h	2.7298	-1.3561	-4.3016
h	2.9089	0.4284	-4.2250
h	1.8585	-0.3327	-5.4446
o	-0.4298	-2.6129	-1.0440
h	0.4427	-2.6618	-1.4648
h	-0.7374	-3.5201	-0.9117
h	-0.4858	0.6254	-2.0497
h	-2.0383	1.0075	-1.9179
h	-3.3153	-1.6257	-0.5043
h	-4.0619	-1.9686	-1.8676
c	-1.6080	-3.7029	-4.6832
h	-1.5319	-2.8401	-5.3586
h	-1.6264	-4.7113	-5.1244
o	-4.2389	-3.4386	-5.4994
h	-4.9630	-3.9005	-5.0537
h	-4.6518	-2.9257	-6.2129

Table 12: B3LYP/6-31++G(d,p) optimized geometry of model TS22

p	-4.2735	-1.3917	-4.5104
o	-5.4354	-1.7627	-3.6202
o	-2.8849	-1.5089	-3.8999
o	-4.4527	-0.4120	-5.6906
mg	-2.2130	-2.2532	-2.1286
o	-2.1496	-4.2380	-3.1863
o	-0.1798	-2.2693	-2.3747
o	-2.1179	-0.4280	-1.1327
o	-4.1902	-2.6600	-1.4876
c	0.6623	-1.3165	-2.1617
o	0.3824	-0.2465	-1.5665
c	2.0780	-1.5350	-2.6651
h	2.7105	-0.6785	-2.4283
h	2.0594	-1.6936	-3.7487
h	2.4962	-2.4410	-2.2131
o	-1.6808	-3.4996	-0.4300
h	-0.7316	-3.6173	-0.5944
h	-2.0875	-4.3766	-0.4383
h	-1.1136	-0.2228	-1.2228
h	-2.5944	0.3124	-1.5292
h	-4.4562	-2.2381	-0.6608
h	-4.8274	-2.3615	-2.2040
c	-1.7466	-4.2993	-4.3371
h	-1.5141	-3.3897	-4.9089
h	-1.6151	-5.2790	-4.8266
o	-4.2955	-2.6706	-5.9565
h	-5.1263	-3.1734	-5.9580
h	-4.4522	-1.5762	-6.3532

Table 13: B3LYP/6-31++G(d,p) optimized geometry of model P2

p	-3.2564	-3.2398	-2.8696
o	-3.2330	-2.4197	-1.5883
o	-1.8535	-3.6194	-3.3975
o	-4.0881	-2.5571	-4.0568
mg	-0.2719	-2.3687	-3.3411
o	0.1699	-3.1287	-5.3600
o	-0.6167	-0.5233	-4.0874
o	-0.7560	-1.7633	-1.3693
o	0.6052	-4.1072	-2.3715
c	-1.5741	-0.0473	-4.8100
o	-2.5304	-0.7222	-5.2496
c	-1.5177	1.4424	-5.1060
h	-2.0817	1.9677	-4.3256
h	-1.9909	1.6609	-6.0655
h	-0.4903	1.8138	-5.0846
o	1.7043	-1.5093	-3.3635
h	1.4741	-0.7142	-3.8778
h	2.4203	-1.9703	-3.8196
h	-0.6588	-0.8201	-1.1871
h	-1.7565	-1.9718	-1.3051
h	-0.2256	-4.6134	-2.4559
h	0.6697	-3.8669	-1.4343
c	-0.7323	-3.4655	-6.1150
h	-1.7769	-3.4829	-5.7787
h	-0.4970	-3.7607	-7.1510
o	-4.0568	-4.6345	-2.6503
h	-3.5651	-1.8369	-4.5035
h	-4.7294	-4.5427	-1.9614

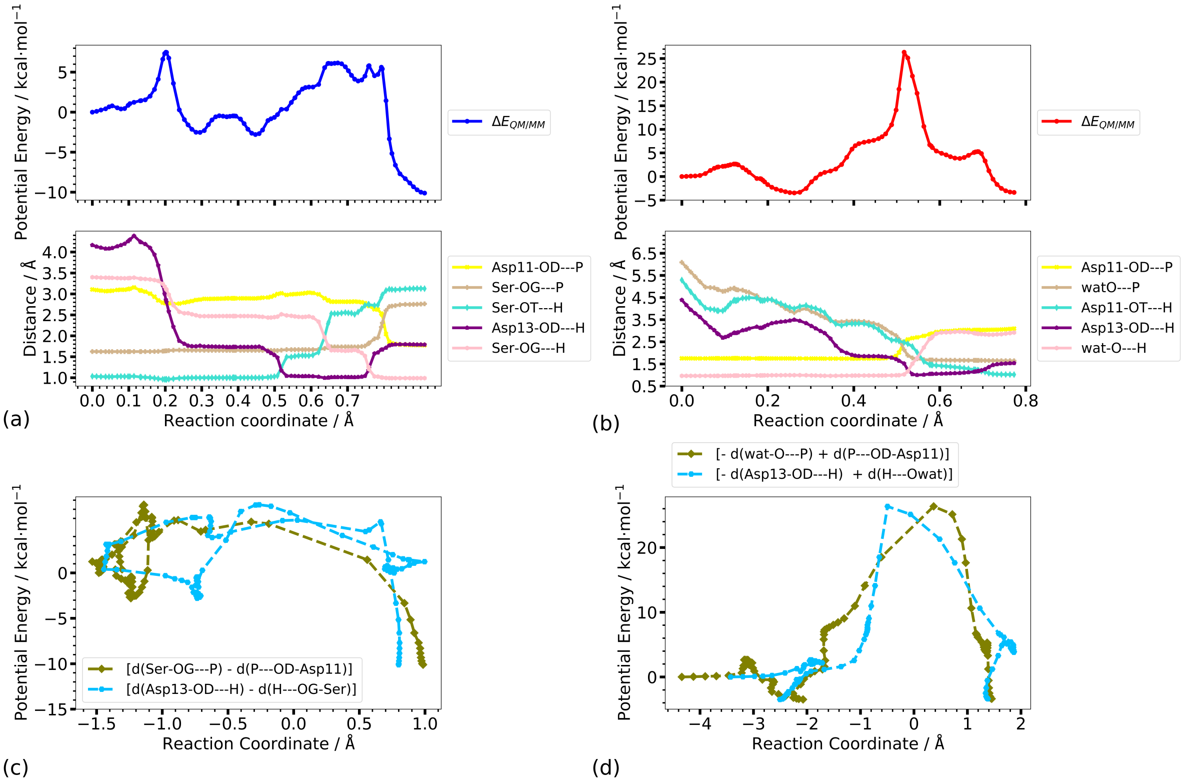

Figure 4: QM/MM minimum energy reaction path profile computed with CPR (top) and important distances (bottom) along the canonical reaction coordinate for (a) Step 1 and (b) Step 2 in the phosphate transfer reaction in phosphoserine phosphatase. QM/MM energy as function of the combined reaction coordinates for P–O bond breaking and formation and proton transfer in (c) Step 1 and (d) Step 2.
